# Genomic data in the All of Us Research Program

**DOI:** 10.1038/s41586-023-06957-x

**Published:** 2024-02-19

**Authors:** The All of Us Research Program Genomics Investigators, Alexander G. Bick, Ginger A. Metcalf, Kelsey R. Mayo, Lee Lichtenstein, Shimon Rura, Robert J. Carroll, Anjene Musick, Jodell E. Linder, I. King Jordan, Shashwat Deepali Nagar, Shivam Sharma, Robert Meller, Melissa Basford, Eric Boerwinkle, Mine S. Cicek, Kimberly F. Doheny, Evan E. Eichler, Stacey Gabriel, Richard A. Gibbs, David Glazer, Paul A. Harris, Gail P. Jarvik, Anthony Philippakis, Heidi L. Rehm, Dan M. Roden, Stephen N. Thibodeau, Scott Topper, Ashley L. Blegen, Samantha J. Wirkus, Victoria A. Wagner, Jeffrey G. Meyer, Mine S. Cicek, Donna M. Muzny, Eric Venner, Michelle Z. Mawhinney, Sean M. L. Griffith, Elvin Hsu, Hua Ling, Marcia K. Adams, Kimberly Walker, Jianhong Hu, Harsha Doddapaneni, Christie L. Kovar, Mullai Murugan, Shannon Dugan, Ziad Khan, Eric Boerwinkle, Niall J. Lennon, Christina Austin-Tse, Eric Banks, Michael Gatzen, Namrata Gupta, Emma Henricks, Katie Larsson, Sheli McDonough, Steven M. Harrison, Christopher Kachulis, Matthew S. Lebo, Cynthia L. Neben, Marcie Steeves, Alicia Y. Zhou, Joshua D. Smith, Christian D. Frazar, Colleen P. Davis, Karynne E. Patterson, Marsha M. Wheeler, Sean McGee, Christina M. Lockwood, Brian H. Shirts, Colin C. Pritchard, Mitzi L. Murray, Valeria Vasta, Dru Leistritz, Matthew A. Richardson, Jillian G. Buchan, Aparna Radhakrishnan, Niklas Krumm, Brenna W. Ehmen, Sophie Schwartz, M. Morgan T. Aster, Kristian Cibulskis, Andrea Haessly, Rebecca Asch, Aurora Cremer, Kylee Degatano, Akum Shergill, Laura D. Gauthier, Samuel K. Lee, Aaron Hatcher, George B. Grant, Genevieve R. Brandt, Miguel Covarrubias, Eric Banks, Ashley Able, Ashley E. Green, Robert J. Carroll, Jennifer Zhang, Henry R. Condon, Yuanyuan Wang, Moira K. Dillon, C. H. Albach, Wail Baalawi, Seung Hoan Choi, Xin Wang, Elisabeth A. Rosenthal, Andrea H. Ramirez, Sokny Lim, Siddhartha Nambiar, Bradley Ozenberger, Anastasia L. Wise, Chris Lunt, Geoffrey S. Ginsburg, Joshua C. Denny

**Affiliations:** 1 Division of Genetic Medicine, Department of Medicine, Vanderbilt University Medical Center, Nashville, TN USA; 2 Human Genome Sequencing Center, Baylor College of Medicine, Houston, TX USA; 3 Vanderbilt Institute of Clinical and Translational Research, Vanderbilt University Medical Center, Nashville, TN USA; 4 Data Sciences Platform, Broad Institute of MIT and Harvard, Cambridge, MA USA; 5 Verily, South San Francisco, CA USA; 6 Department of Biomedical Informatics, Vanderbilt University Medical Center, Nashville, TN USA; 7 All of Us Research Program, National Institutes of Health, Bethesda, MD USA; 8 School of Biological Sciences, Georgia Institute of Technology, Atlanta, GA USA; 9 Neuroscience Institute, Institute of Translational Genomic Medicine, Morehouse School of Medicine, Atlanta, GA USA; 10 Department of Laboratory Medicine and Pathology, Mayo Clinic, Rochester, MN USA; 11 Department of Genetic Medicine, Johns Hopkins University School of Medicine, Baltimore, MD USA; 12 Department of Genome Sciences, University of Washington School of Medicine, Seattle, WA USA; 13 Howard Hughes Medical Institute, University of Washington, Seattle, WA USA; 14 Broad Institute of MIT and Harvard, Cambridge, MA USA; 15 Division of Medical Genetics, Department of Medicine, University of Washington School of Medicine, Seattle, WA USA; 16 Department of Medicine, Vanderbilt University Medical Center, Nashville, TN USA; 17 Department of Pharmacology, Vanderbilt University Medical Center, Nashville, TN USA; 18 Center for Individualized Medicine, Biorepository Program, Mayo Clinic, Rochester, MN USA; 19 Color Health, Burlingame, CA USA; 20 School of Public Health, University of Texas Health Science Center at Houston, Houston, TX USA; 21 Laboratory for Molecular Medicine, Massachusetts General Brigham Personalized Medicine, Cambridge, MA USA; 22 Department of Laboratory Medicine and Pathology, University of Washington School of Medicine, Seattle, WA USA

**Keywords:** Genomics, Sequencing, Databases, Genome-wide association studies, Genetic variation

## Abstract

Comprehensively mapping the genetic basis of human disease across diverse individuals is a long-standing goal for the field of human genetics^1–4^. The All of Us Research Program is a longitudinal cohort study aiming to enrol a diverse group of at least one million individuals across the USA to accelerate biomedical research and improve human health^5,6^. Here we describe the programme’s genomics data release of 245,388 clinical-grade genome sequences. This resource is unique in its diversity as 77% of participants are from communities that are historically under-represented in biomedical research and 46% are individuals from under-represented racial and ethnic minorities. All of Us identified more than 1 billion genetic variants, including more than 275 million previously unreported genetic variants, more than 3.9 million of which had coding consequences. Leveraging linkage between genomic data and the longitudinal electronic health record, we evaluated 3,724 genetic variants associated with 117 diseases and found high replication rates across both participants of European ancestry and participants of African ancestry. Summary-level data are publicly available, and individual-level data can be accessed by researchers through the All of Us Researcher Workbench using a unique data passport model with a median time from initial researcher registration to data access of 29 hours. We anticipate that this diverse dataset will advance the promise of genomic medicine for all.

## Abstract (web summary)

A study describes the release of clinical-grade whole-genome sequence data for 245,388 diverse participants by the All of Us Research Program and characterizes the properties of the dataset.

## Main

Comprehensively identifying genetic variation and cataloguing its contribution to health and disease, in conjunction with environmental and lifestyle factors, is a central goal of human health research^1,2^. A key limitation in efforts to build this catalogue has been the historic under-representation of large subsets of individuals in biomedical research including individuals from diverse ancestries, individuals with disabilities and individuals from disadvantaged backgrounds^3,4^. The All of Us Research Program (All of Us) aims to address this gap by enrolling and collecting comprehensive health data on at least one million individuals who reflect the diversity across the USA^5,6^. An essential component of All of Us is the generation of whole-genome sequence (WGS) and genotyping data on one million participants. All of Us is committed to making this dataset broadly useful—not only by democratizing access to this dataset across the scientific community but also to return value to the participants themselves by returning individual DNA results, such as genetic ancestry, hereditary disease risk and pharmacogenetics according to clinical standards, to those who wish to receive these research results.

Here we describe the release of WGS data from 245,388 All of Us participants and demonstrate the impact of this high-quality data in genetic and health studies. We carried out a series of data harmonization and quality control (QC) procedures and conducted analyses characterizing the properties of the dataset including genetic ancestry and relatedness. We validated the data by replicating well-established genotype–phenotype associations including low-density lipoprotein cholesterol (LDL-C) and 117 additional diseases. These data are available through the All of Us Researcher Workbench, a cloud platform that embodies and enables programme priorities, facilitating equitable data and compute access while ensuring responsible conduct of research and protecting participant privacy through a passport data access model.

## The All of Us Research Program

**Fig. 1:**
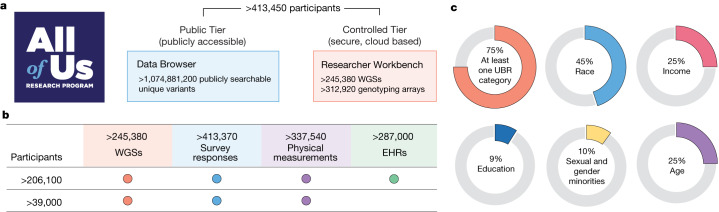
Summary of All of Us data resources. **a**, The All of Us Research Hub contains a publicly accessible Data Browser for exploration of summary phenotypic and genomic data. The Researcher Workbench is a secure cloud-based environment of participant-level data in a Controlled Tier that is widely accessible to researchers. **b**, All of Us participants have rich phenotype data from a combination of physical measurements, survey responses, EHRs, wearables and genomic data. Dots indicate the presence of the specific data type for the given number of participants. **c**, Overall summary of participants under-represented in biomedical research (UBR) with data available in the Controlled Tier. The All of Us logo in **a** is reproduced with permission of the National Institutes of Health’s All of Us Research Program.

To accelerate health research, All of Us is committed to curating and releasing research data early and often^6^. Less than five years after national enrolment began in 2018, this fifth data release includes data from more than 413,000 All of Us participants. Summary data are made available through a public Data Browser, and individual-level participant data are made available to researchers through the Researcher Workbench (Fig. 1a and Data availability).

**Extended Data Fig. 1:**
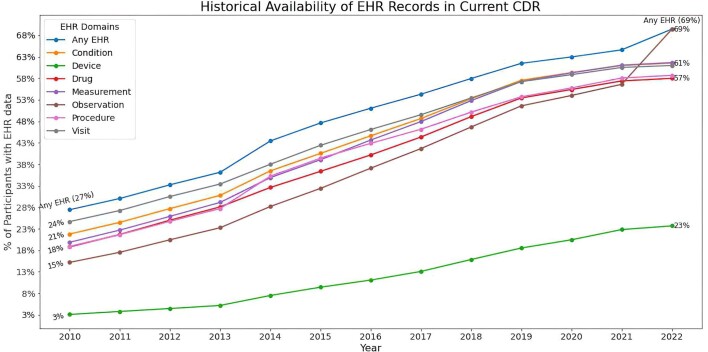
Historic availability of EHR records in All of Us v7 Controlled Tier Curated Data Repository (N = 413,457). For better visibility, the plot shows growth starting in 2010.

Participant data include a rich combination of phenotypic and genomic data (Fig. 1b). Participants are asked to complete consent for research use of data, sharing of electronic health records (EHRs), donation of biospecimens (blood or saliva, and urine), in-person provision of physical measurements (height, weight and blood pressure) and surveys initially covering demographics, lifestyle and overall health^7^. Participants are also consented for recontact. EHR data, harmonized using the Observational Medical Outcomes Partnership Common Data Model^8^ (Methods), are available for more than 287,000 participants (69.42%) from more than 50 health care provider organizations. The EHR dataset is longitudinal, with a quarter of participants having 10 years of EHR data (Extended Data Fig. 1). Data include 245,388 WGSs and genome-wide genotyping on 312,925 participants. Sequenced and genotyped individuals in this data release were not prioritized on the basis of any clinical or phenotypic feature. Notably, 99% of participants with WGS data also have survey data and physical measurements, and 84% also have EHR data. In this data release, 77% of individuals with genomic data identify with groups historically under-represented in biomedical research, including 46% who self-identify with a racial or ethnic minority group (Fig. 1c, Supplementary Table 1 and Supplementary Note).

## Scaling the All of Us infrastructure

The genomic dataset generated from All of Us participants is a resource for research and discovery and serves as the basis for return of individual health-related DNA results to participants. Consequently, the US Food and Drug Administration determined that All of Us met the criteria for a significant risk device study. As such, the entire All of Us genomics effort from sample acquisition to sequencing meets clinical laboratory standards^9^.

All of Us participants were recruited through a national network of partners, starting in 2018, as previously described^5^. Participants may enrol through All of Us*-*funded health care provider organizations or direct volunteer pathways and all biospecimens, including blood and saliva, are sent to the central All of Us Biobank for processing and storage. Genomics data for this release were generated from blood-derived DNA. The programme began return of actionable genomic results in December 2022. As of April 2023, approximately 51,000 individuals were sent notifications asking whether they wanted to view their results, and approximately half have accepted. Return continues on an ongoing basis.

The All of Us Data and Research Center maintains all participant information and biospecimen ID linkage to ensure that participant confidentiality and coded identifiers (participant and aliquot level) are used to track each sample through the All of Us genomics workflow. This workflow facilitates weekly automated aliquot and plating requests to the Biobank, supplies relevant metadata for the sample shipments to the Genome Centers, and contains a feedback loop to inform action on samples that fail QC at any stage. Further, the consent status of each participant is checked before sample shipment to confirm that they are still active. Although all participants with genomic data are consented for the same general research use category, the programme accommodates different preferences for the return of genomic data to participants and only data for those individuals who have consented for return of individual health-related DNA results are distributed to the All of Us Clinical Validation Labs for further evaluation and health-related clinical reporting. All participants in All of Us that choose to get health-related DNA results have the option to schedule a genetic counselling appointment to discuss their results. Individuals with positive findings who choose to obtain results are required to schedule an appointment with a genetic counsellor to receive those findings.

## Genome sequencing

To satisfy the requirements for clinical accuracy, precision and consistency across DNA sample extraction and sequencing, the All of Us Genome Centers and Biobank harmonized laboratory protocols, established standard QC methodologies and metrics, and conducted a series of validation experiments using previously characterized clinical samples and commercially available reference standards^9^. Briefly, PCR-free barcoded WGS libraries were constructed with the Illumina Kapa HyperPrep kit. Libraries were pooled and sequenced on the Illumina NovaSeq 6000 instrument. After demultiplexing, initial QC analysis is performed with the Illumina DRAGEN pipeline (Supplementary Table 2) leveraging lane, library, flow cell, barcode and sample level metrics as well as assessing contamination, mapping quality and concordance to genotyping array data independently processed from a different aliquot of DNA. The Genome Centers use these metrics to determine whether each sample meets programme specifications and then submits sequencing data to the Data and Research Center for further QC, joint calling and distribution to the research community (Methods).

This effort to harmonize sequencing methods, multi-level QC and use of identical data processing protocols mitigated the variability in sequencing location and protocols that often leads to batch effects in large genomic datasets^9^. As a result, the data are not only of clinical-grade quality, but also consistent in coverage (≥30× mean) and uniformity across Genome Centers (Supplementary Figs. 1–5).

## Joint calling and variant discovery

**Extended Data Fig. 2:**
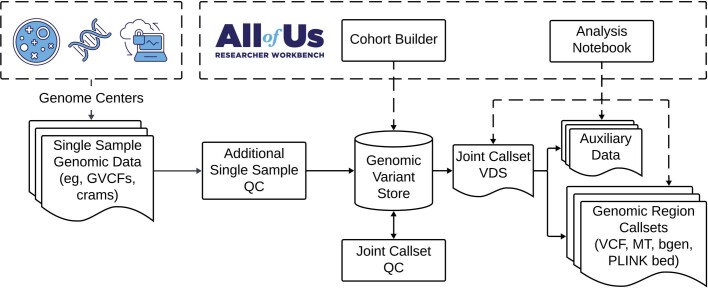
Overview of the Genomic Data Curation Pipeline for WGS samples. The Data and Research Center (DRC) performs additional single sample quality control (QC) on the data as it arrives from the Genome Centers. The variants from samples that pass this QC are loaded into the Genomic Variant Store (GVS), where we jointly call the variants and apply additional QC. We apply a joint call set QC process, which is stored with the call set. The entire joint call set is rendered as a Hail Variant Dataset (VDS), which can be accessed from the analysis notebooks in the Researcher Workbench. Subsections of the genome are extracted from the VDS and rendered in different formats with all participants. Auxiliary data can also be accessed through the Researcher Workbench. This includes variant functional annotations, joint call set QC results, predicted ancestry, and relatedness. Auxiliary data are derived from GVS (arrow not shown) and the VDS. The Cohort Builder directly queries GVS when researchers request genomic data for subsets of samples. Aligned reads, as cram files, are available in the Researcher Workbench (not shown). The graphics of the dish, gene and computer and the All of Us logo are reproduced with permission of the National Institutes of Health’s All of Us Research Program.

We carried out joint calling across the entire All of Us WGS dataset (Extended Data Fig. 2). Joint calling leverages information across samples to prune artefact variants, which increases sensitivity, and enables flagging samples with potential issues that were missed during single-sample QC^10^ (Supplementary Table 3). Scaling conventional approaches to whole-genome joint calling beyond 50,000 individuals is a notable computational challenge^11,12^. To address this, we developed a new cloud variant storage solution, the Genomic Variant Store (GVS), which is based on a schema designed for querying and rendering variants in which the variants are stored in GVS and rendered to an analysable variant file, as opposed to the variant file being the primary storage mechanism (Code availability). We carried out QC on the joint call set on the basis of the approach developed for gnomAD 3.1 (ref. ^13^). This included flagging samples with outlying values in eight metrics (Supplementary Table 4, Supplementary Fig. 2 and Methods).

To calculate the sensitivity and precision of the joint call dataset, we included four well-characterized samples. We sequenced the National Institute of Standards and Technology reference materials (DNA samples) from the Genome in a Bottle consortium^13^ and carried out variant calling as described above. We used the corresponding published set of variant calls for each sample as the ground truth in our sensitivity and precision calculations^14^. The overall sensitivity for single-nucleotide variants was over 98.7% and precision was more than 99.9%. For short insertions or deletions, the sensitivity was over 97% and precision was more than 99.6% (Supplementary Table 5 and Methods).

**Extended Data Fig. 3:**
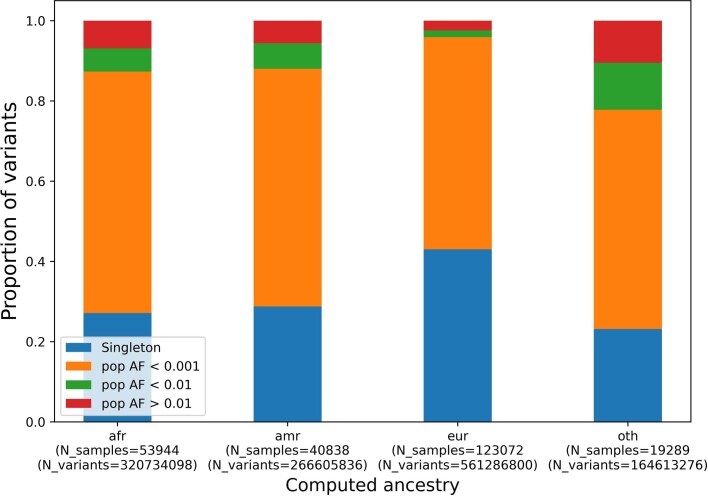
Proportion of allelic frequencies (AF), stratified by computed ancestry with over 10,000 participants. Bar counts are not cumulative (eg, “pop AF < 0.01” does not include “pop AF < 0.001”).

**Extended Data Fig. 4:**
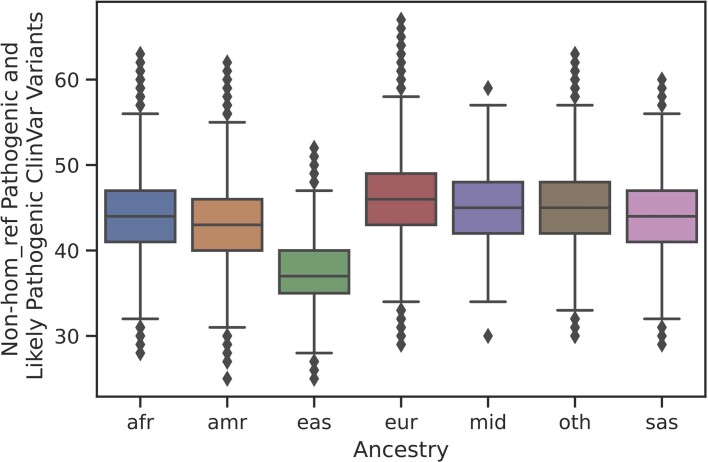
Distribution of pathogenic, and likely pathogenic ClinVar variants. Stratified by ancestry filtered to only those variants that are found in allele count (AC) < 40 individuals for 245,388 short read WGS samples.

The joint call set included more than 1 billion genetic variants. We annotated the joint call dataset on the basis of functional annotation (for example, gene symbol and protein change) using Illumina Nirvana^15^. We defined coding variants as those inducing an amino acid change on a canonical ENSEMBL transcript and found 272,051,104 non-coding and 3,913,722 coding variants that have not been described previously in dbSNP^16^ v153 (Extended Data Table 1). A total of 3,912,832 (99.98%) of the coding variants are rare (allelic frequency < 0.01) and the remaining 883 (0.02%) are common (allelic frequency > 0.01). Of the coding variants, 454 (0.01%) are common in one or more of the non-European computed ancestries in All of Us, rare among participants of European ancestry, and have an allelic number greater than 1,000 (Extended Data Table 2 and Extended Data Fig. 3). The distributions of pathogenic, or likely pathogenic, ClinVar variant counts per participant, stratified by computed ancestry, filtered to only those variants that are found in individuals with an allele count of <40 are shown in Extended Data Fig. 4. The potential medical implications of these known and new variants with respect to variant pathogenicity by ancestry are highlighted in a companion paper^17^. In particular, we find that the European ancestry subset has the highest rate of pathogenic variation (2.1%), which was twice the rate of pathogenic variation in individuals of East Asian ancestry^17^ .The lower frequency of variants in East Asian individuals may be partially explained by the fact the sample size in that group is small and there may be knowledge bias in the variant databases that is reducing the number of findings in some of the less-studied ancestry groups.

## Genetic ancestry and relatedness

**Fig. 2:**
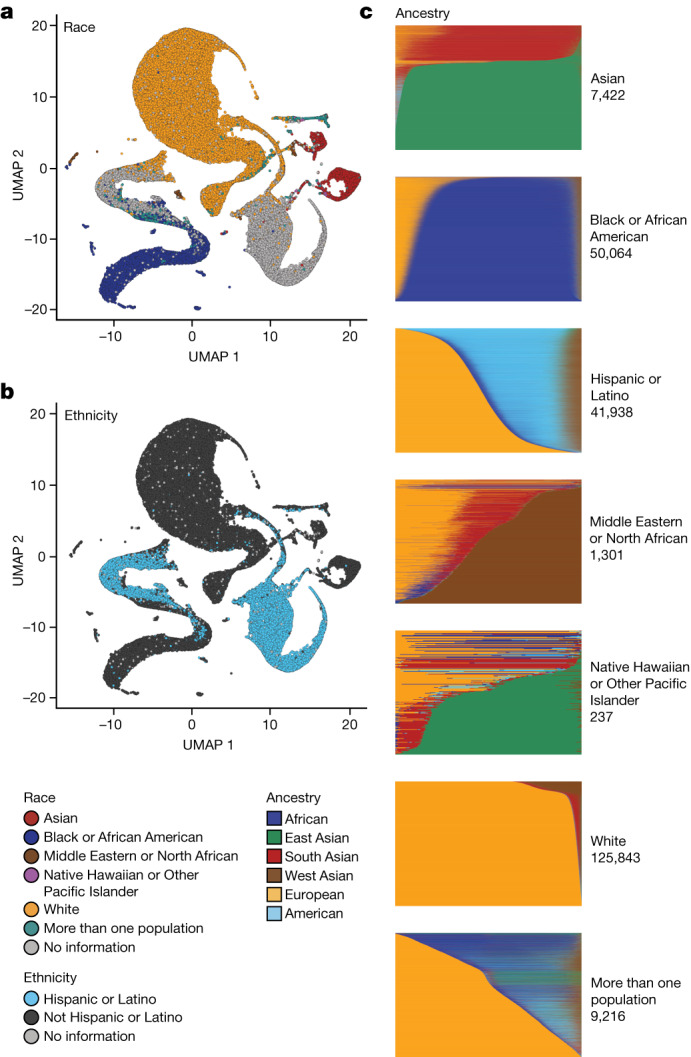
Genetic ancestry in All of Us. **a**,**b**, Uniform manifold approximation and projection (UMAP) representations of All of Us WGS PCA data with self-described race (**a**) and ethnicity (**b**) labels. **c**, Proportion of genetic ancestry per individual in six distinct and coherent ancestry groups defined by Human Genome Diversity Project and 1000 Genomes samples.

Genetic ancestry inference confirmed that 51.1% of the All of Us WGS dataset is derived from individuals of non-European ancestry. Briefly, the ancestry categories are based on the same labels used in gnomAD^18^. We trained a classifier on a 16-dimensional principal component analysis (PCA) space of a diverse reference based on 3,202 samples and 151,159 autosomal single-nucleotide polymorphisms. We projected the All of Us samples into the PCA space of the training data, based on the same single-nucleotide polymorphisms from the WGS data, and generated categorical ancestry predictions from the trained classifier (Methods). Continuous genetic ancestry fractions for All of Us samples were inferred using the same PCA data, and participants’ patterns of ancestry and admixture were compared to their self-identified race and ethnicity (Fig. 2 and Methods). Continuous ancestry inference carried out using genome-wide genotypes yields highly concordant estimates.

Kinship estimation confirmed that All of Us WGS data consist largely of unrelated individuals with about 85% (215,107) having no first- or second-degree relatives in the dataset (Supplementary Fig. 6). As many genomic analyses leverage unrelated individuals, we identified the smallest set of samples that are required to be removed from the remaining individuals that had first- or second-degree relatives and retained one individual from each kindred. This procedure yielded a maximal independent set of 231,442 individuals (about 94%) with genome sequence data in the current release (Methods).

## Genetic determinants of LDL-C

**Fig. 3:**
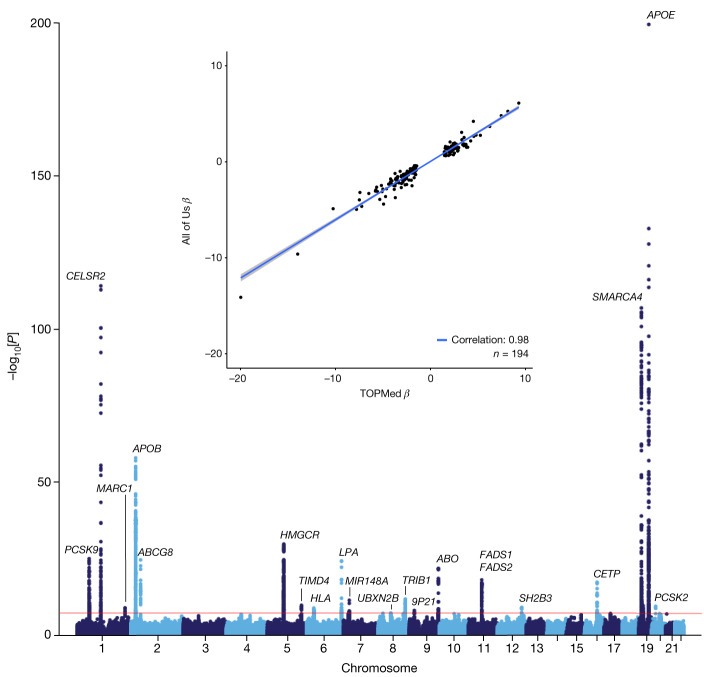
All of Us LDL-C GWAS. Manhattan plot demonstrating robust replication of 20 well-established LDL-C genetic loci among 91,749 individuals with 1 or more LDL-C measurements. The red horizontal line denotes the genome wide significance threshold of *P* = 5 × 10^–8^. Inset, effect estimate (*β*) comparison between NHLBI TOPMed LDL-C GWAS (*x* axis) and All of Us LDL-C GWAS (*y* axis) for the subset of 194 independent variants clumped (window 250 kb, r2 0.5) that reached genome-wide significance in NHLBI TOPMed.

As a measure of data quality and utility, we carried out a single-variant genome-wide association study (GWAS) for LDL-C, a trait with well-established genomic architecture (Methods). Of the 245,388 WGS participants, 91,749 had one or more LDL-C measurements. The All of Us LDL-C GWAS identified 20 well-established genome-wide significant loci, with minimal genomic inflation (Fig. 3, Extended Data Table 3 and Supplementary Fig. 7). We compared the results to those of a recent multi-ethnic LDL-C GWAS in the National Heart, Lung, and Blood Institute (NHLBI) TOPMed study that included 66,329 ancestrally diverse (56% non-European ancestry) individuals^19^. We found a strong correlation between the effect estimates for NHLBI TOPMed genome-wide significant loci and those of All of Us (*R*^2^ = 0.98, *P* < 1.61 × 10^−45^; Fig. 3, inset). Notably, the per-locus effect sizes observed in All of Us are decreased compared to those in TOPMed, which is in part due to differences in the underlying statistical model, differences in the ancestral composition of these datasets and differences in laboratory value ascertainment between EHR-derived data and epidemiology studies. A companion manuscript extended this work to identify common and rare genetic associations for three diseases (atrial fibrillation, coronary artery disease and type 2 diabetes) and two quantitative traits (height and LDL-C) in the All of Us dataset and identified very high concordance with previous efforts across all of these diseases and traits^20^.

## Genotype-by-phenotype associations

As another measure of data quality and utility, we tested replication rates of previously reported phenotype–genotype associations in the five predicted genetic ancestry populations present in the Phenotype/Genotype Reference Map (PGRM): AFR, African ancestry; AMR, Latino/admixed American ancestry; EAS, East Asian ancestry; EUR, European ancestry; SAS, South Asian ancestry. The PGRM contains published associations in the GWAS catalogue in these ancestry populations that map to International Classification of Diseases-based phenotype codes^21^. This replication study specifically looked across 4,947 variants, calculating replication rates for powered associations in each ancestry population. The overall replication rates for associations powered at 80% were: 72.0% (18/25) in AFR, 100% (13/13) in AMR, 46.6% (7/15) in EAS, 74.9% (1,064/1,421) in EUR, and 100% (1/1) in SAS. With the exception of the EAS ancestry results, these powered replication rates are comparable to those of the published PGRM analysis where the replication rates of several single-site EHR-linked biobanks ranges from 76% to 85%. These results demonstrate the utility of the data and also highlight opportunities for further work understanding the specifics of the All of Us population and the potential contribution of gene–environment interactions to genotype–phenotype mapping and motivates the development of methods for multi-site EHR phenotype data extraction, harmonization and genetic association studies.

**Fig. 4:**
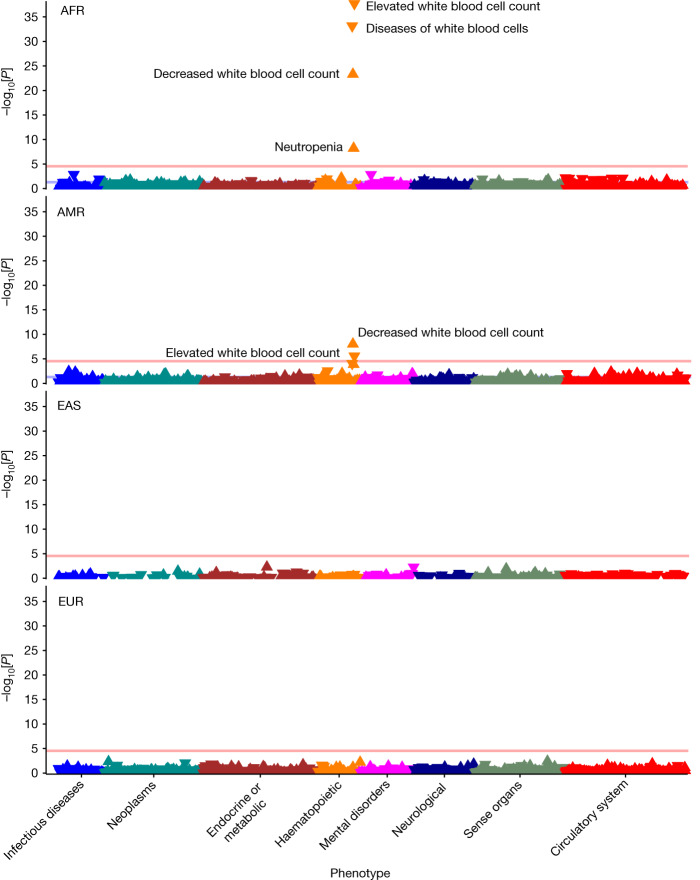
Phenome-wide associations of the Duffy blood group locus (rs2814778, *ACKR1*). Results of genetic-ancestry-stratified phenome-wide association analysis among unrelated individuals highlighting ancestry-specific disease associations across the four most common genetic ancestries of participant. Bonferroni-adjusted phenome-wide significance threshold (<2.88 × 10^−5^) is plotted as a red horizontal line. AFR (*n* = 34,037, minor allele fraction (MAF) 0.82); AMR (*n* = 28,901, MAF 0.10); EAS (*n* = 32,55, MAF 0.003); EUR (*n* = 101,613, MAF 0.007).

**Extended Data Fig. 5:**
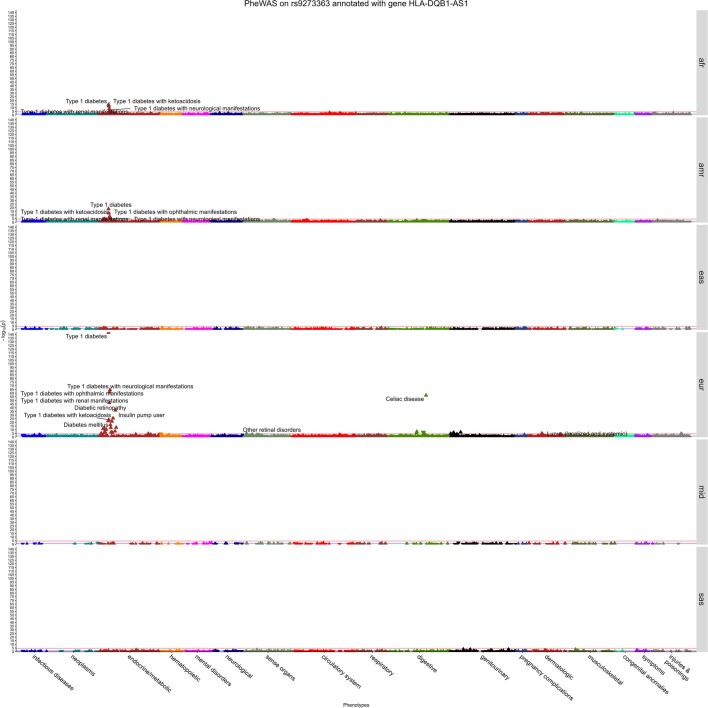
Ancestry specific HLA-DQB1 (rs9273363) locus associations in 231,442 unrelated individuals. Phenome-wide (PheWAS) associations highlight ancestry specific consequences across ancestries.

**Extended Data Fig. 6:**
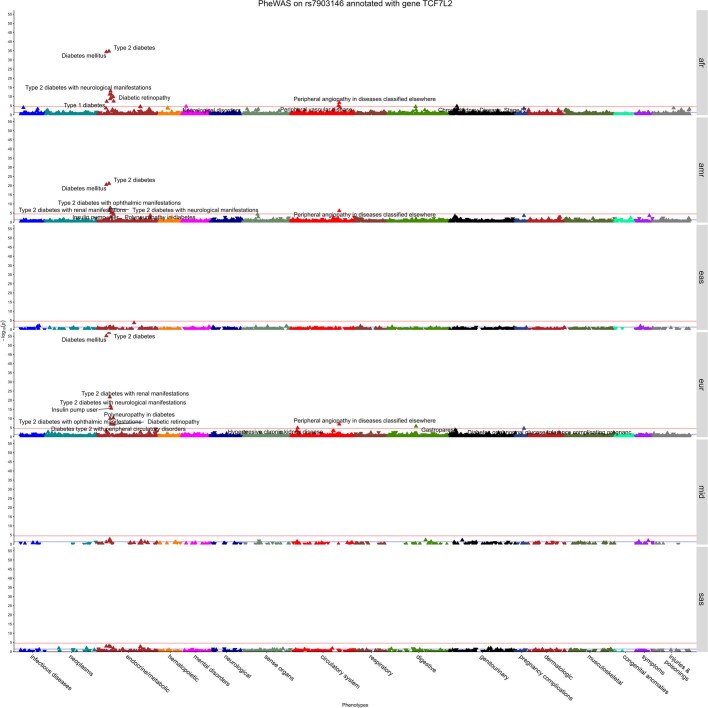
Ancestry specific TCF7L2 (rs7903146) locus associations in 231,442 unrelated individuals. Phenome-wide (PheWAS) associations highlight diabetic consequences across ancestries.

More broadly, the All of Us resource highlights the opportunities to identify genotype–phenotype associations that differ across diverse populations^22^. For example, the Duffy blood group locus (*ACKR1*) is more prevalent in individuals of AFR ancestry and individuals of AMR ancestry than in individuals of EUR ancestry. Although the phenome-wide association study of this locus highlights the well-established association of the Duffy blood group with lower white blood cell counts both in individuals of AFR and AMR ancestry^23,24^, it also revealed genetic-ancestry-specific phenotype patterns, with minimal phenotypic associations in individuals of EAS ancestry and individuals of EUR ancestry (Fig. 4 and Extended Data Table 4). Conversely, rs9273363 in the *HLA-DQB1* locus is associated with increased risk of type 1 diabetes^25,26^ and diabetic complications across ancestries, but only associates with increased risk of coeliac disease in individuals of EUR ancestry (Extended Data Fig. 5). Similarly, the *TCF7L2* locus^27^ strongly associates with increased risk of type 2 diabetes and associated complications across several ancestries (Extended Data Fig. 6). Association testing results are available in Supplementary Dataset 1.

## The cloud-based Researcher Workbench

All of Us genomic data are available in a secure, access-controlled cloud-based analysis environment: the All of Us Researcher Workbench. Unlike traditional data access models that require per-project approval, access in the Researcher Workbench is governed by a data passport model based on a researcher’s authenticated identity, institutional affiliation, and completion of self-service training and compliance attestation^28^. After gaining access, a researcher may create a new workspace at any time to conduct a study, provided that they comply with all Data Use Policies and self-declare their research purpose. This information is regularly audited and made accessible publicly on the All of Us Research Projects Directory. This streamlined access model is guided by the principles that: participants are research partners and maintaining their privacy and data security is paramount; their data should be made as accessible as possible for authorized researchers; and we should continually seek to remove unnecessary barriers to accessing and using All of Us data.

For researchers at institutions with an existing institutional data use agreement, access can be gained as soon as they complete the required verification and compliance steps. As of August 2023, 556 institutions have agreements in place, allowing more than 5,000 approved researchers to actively work on more than 4,400 projects. The median time for a researcher from initial registration to completion of these requirements is 28.6 h (10th percentile: 48 min, 90th percentile: 14.9 days), a fraction of the weeks to months it can take to assemble a project-specific application and have it reviewed by an access board with conventional access models.

Given that the size of the project’s phenotypic and genomic dataset is expected to reach 4.75 PB in 2023, the use of a central data store and cloud analysis tools will save funders an estimated US$16.5 million per year when compared to the typical approach of allowing researchers to download genomic data. Storing one copy per institution of this data at 556 registered institutions would cost about US$1.16 billion per year. By contrast, storing a central cloud copy costs about US$1.14 million per year, a 99.9% saving. Importantly, cloud infrastructure also democratizes data access particularly for researchers who do not have high-performance local compute resources.

## Discussion

Here we present the All of Us Research Program’s approach to generating diverse clinical-grade genomic data at an unprecedented scale. We present the data release of about 245,000 genome sequences as part of a scalable framework that will grow to include genetic information and health data for one million or more people living across the USA. Our observations permit several conclusions.

First, the All of Us programme is making a notable contribution to improving the study of human biology through purposeful inclusion of under-represented individuals at scale^29,30^. Of the participants with genomic data in All of Us, 45.92% self-identified as a non-European race or ethnicity. This diversity enabled identification of more than 275 million new genetic variants across the dataset not previously captured by other large-scale genome aggregation efforts with diverse participants that have submitted variation to dbSNP v153, such as NHLBI TOPMed^31^ freeze 8 (Extended Data Table 1). In contrast to gnomAD, All of Us permits individual-level genotype access with detailed phenotype data for all participants. Furthermore, unlike many genomics resources, All of Us is uniformly consented for general research use and enables researchers to go from initial account creation to individual-level data access in as little as a few hours. The All of Us cohort is significantly more diverse than those of other large contemporary research studies generating WGS data^32,33^. This enables a more equitable future for precision medicine (for example, through constructing polygenic risk scores that are appropriately calibrated to diverse populations^34,35^ as the eMERGE programme has done leveraging All of Us data^36,37^). Developing new tools and regulatory frameworks to enable analyses across multiple biobanks in the cloud to harness the unique strengths of each is an active area of investigation addressed in a companion paper to this work^38^.

Second, the All of Us Researcher Workbench embodies the programme’s design philosophy of open science, reproducible research, equitable access and transparency to researchers and to research participants^26^. Importantly, for research studies, no group of data users should have privileged access to All of Us resources based on anything other than data protection criteria. Although the All of Us Researcher Workbench initially targeted onboarding US academic, health care and non-profit organizations, it has recently expanded to international researchers. We anticipate further genomic and phenotypic data releases at regular intervals with data available to all researcher communities. We also anticipate additional derived data and functionality to be made available, such as reference data, structural variants and a service for array imputation using the All of Us genomic data.

Third, All of Us enables studying human biology at an unprecedented scale. The programmatic goal of sequencing one million or more genomes has required harnessing the output of multiple sequencing centres. Previous work has focused on achieving functional equivalence in data processing and joint calling pipelines^39^. To achieve clinical-grade data equivalence, All of Us required protocol equivalence at both sequencing production level and data processing across the sequencing centres. Furthermore, previous work has demonstrated the value of joint calling at scale^10,18^. The new GVS framework developed by the All of Us programme enables joint calling at extreme scales (Code availability). Finally, the provision of data access through cloud-native tools enables scalable and secure access and analysis to researchers while simultaneously enabling the trust of research participants and transparency underlying the All of Us data passport access model.

The clinical-grade sequencing carried out by All of Us enables not only research, but also the return of value to participants through clinically relevant genetic results and health-related traits to those who opt-in to receiving this information. In the years ahead, we anticipate that this partnership with All of Us participants will enable researchers to move beyond large-scale genomic discovery to understanding the consequences of implementing genomic medicine at scale.

## Methods

### The All of Us cohort

All of Us aims to engage a longitudinal cohort of one million or more US participants, with a focus on including populations that have historically been under-represented in biomedical research. Details of the All of Us cohort have been described previously^5^. Briefly, the primary objective is to build a robust research resource that can facilitate the exploration of biological, clinical, social and environmental determinants of health and disease. The programme will collect and curate health-related data and biospecimens, and these data and biospecimens will be made broadly available for research uses. Health data are obtained through the electronic medical record and through participant surveys. Survey templates can be found on our public website: https://www.researchallofus.org/data-tools/survey-explorer/. Adults 18 years and older who have the capacity to consent and reside in the USA or a US territory at present are eligible. Informed consent for all participants is conducted in person or through an eConsent platform that includes primary consent, HIPAA Authorization for Research use of EHRs and other external health data, and Consent for Return of Genomic Results. The protocol was reviewed by the Institutional Review Board (IRB) of the All of Us Research Program. The All of Us IRB follows the regulations and guidance of the NIH Office for Human Research Protections for all studies, ensuring that the rights and welfare of research participants are overseen and protected uniformly.

### Data accessibility through a ‘data passport’

Authorization for access to participant-level data in All of Us is based on a ‘data passport’ model, through which authorized researchers do not need IRB review for each research project. The data passport is required for gaining data access to the Researcher Workbench and for creating workspaces to carry out research projects using All of Us data. At present, data passports are authorized through a six-step process that includes affiliation with an institution that has signed a Data Use and Registration Agreement, account creation, identity verification, completion of ethics training, and attestation to a data user code of conduct. Results reported follow the All of Us Data and Statistics Dissemination Policy disallowing disclosure of group counts under 20 to protect participant privacy without seeking prior approval^40^.

### EHR data

At present, All of Us gathers EHR data from about 50 health care organizations that are funded to recruit and enrol participants as well as transfer EHR data for those participants who have consented to provide them. Data stewards at each provider organization harmonize their local data to the Observational Medical Outcomes Partnership (OMOP) Common Data Model, and then submit it to the All of Us Data and Research Center (DRC) so that it can be linked with other participant data and further curated for research use. OMOP is a common data model standardizing health information from disparate EHRs to common vocabularies and organized into tables according to data domains. EHR data are updated from the recruitment sites and sent to the DRC quarterly. Updated data releases to the research community occur approximately once a year. Supplementary Table 6 outlines the OMOP concepts collected by the DRC quarterly from the recruitment sites.

### Biospecimen collection and processing

Participants who consented to participate in All of Us donated fresh whole blood (4 ml EDTA and 10 ml EDTA) as a primary source of DNA. The All of Us Biobank managed by the Mayo Clinic extracted DNA from 4 ml EDTA whole blood, and DNA was stored at −80 °C at an average concentration of 150 ng µl^−1^. The buffy coat isolated from 10 ml EDTA whole blood has been used for extracting DNA in the case of initial extraction failure or absence of 4 ml EDTA whole blood. The Biobank plated 2.4 µg DNA with a concentration of 60 ng µl^−1^ in duplicate for array and WGS samples. The samples are distributed to All of Us Genome Centers weekly, and a negative (empty well) control and National Institute of Standards and Technology controls are incorporated every two months for QC purposes.

### Genome sequencing

#### Genome Center sample receipt, accession and QC

On receipt of DNA sample shipments, the All of Us Genome Centers carry out an inspection of the packaging and sample containers to ensure that sample integrity has not been compromised during transport and to verify that the sample containers correspond to the shipping manifest. QC of the submitted samples also includes DNA quantification, using routine procedures to confirm volume and concentration (Supplementary Table 7). Any issues or discrepancies are recorded, and affected samples are put on hold until resolved. Samples that meet quality thresholds are accessioned in the Laboratory Information Management System, and sample aliquots are prepared for library construction processing (for example, normalized with respect to concentration and volume).

#### WGS library construction, sequencing and primary data QC

The DNA sample is first sheared using a Covaris sonicator and is then size-selected using AMPure XP beads to restrict the range of library insert sizes. Using the PCR Free Kapa HyperPrep library construction kit, enzymatic steps are completed to repair the jagged ends of DNA fragments, add proper A-base segments, and ligate indexed adapter barcode sequences onto samples. Excess adaptors are removed using AMPure XP beads for a final clean-up. Libraries are quantified using quantitative PCR with the Illumina Kapa DNA Quantification Kit and then normalized and pooled for sequencing (Supplementary Table 7).

Pooled libraries are loaded on the Illumina NovaSeq 6000 instrument. The data from the initial sequencing run are used to QC individual libraries and to remove non-conforming samples from the pipeline. The data are also used to calibrate the pooling volume of each individual library and re-pool the libraries for additional NovaSeq sequencing to reach an average coverage of 30×.

After demultiplexing, WGS analysis occurs on the Illumina DRAGEN platform. The DRAGEN pipeline consists of highly optimized algorithms for mapping, aligning, sorting, duplicate marking and haplotype variant calling and makes use of platform features such as compression and BCL conversion. Alignment uses the GRCh38dh reference genome. QC data are collected at every stage of the analysis protocol, providing high-resolution metrics required to ensure data consistency for large-scale multiplexing. The DRAGEN pipeline produces a large number of metrics that cover lane, library, flow cell, barcode and sample-level metrics for all runs as well as assessing contamination and mapping quality. The All of Us Genome Centers use these metrics to determine pass or fail for each sample before submitting the CRAM files to the All of Us DRC. For mapping and variant calling, all Genome Centers have harmonized on a set of DRAGEN parameters, which ensures consistency in processing (Supplementary Table 2).

Every step through the WGS procedure is rigorously controlled by predefined QC measures. Various control mechanisms and acceptance criteria were established during WGS assay validation. Specific metrics for reviewing and releasing genome data are: mean coverage (threshold of ≥30×), genome coverage (threshold of ≥90% at 20×), coverage of hereditary disease risk genes (threshold of ≥95% at 20×), aligned Q30 bases (threshold of ≥8 × 10^10^), contamination (threshold of ≤1%) and concordance to independently processed array data.

### Array genotyping

Samples are processed for genotyping at three All of Us Genome Centers (Broad, Johns Hopkins University and University of Washington). DNA samples are received from the Biobank and the process is facilitated by the All of Us genomics workflow described above. All three centres used an identical array product, scanners, resource files and genotype calling software for array processing to reduce batch effects. Each centre has its own Laboratory Information Management System that manages workflow control, sample and reagent tracking, and centre-specific liquid handling robotics.

Samples are processed using the Illumina Global Diversity Array (GDA) with Illumina Infinium LCG chemistry using the automated protocol and scanned on Illumina iSCANs with Automated Array Loaders. Illumina IAAP software converts raw data (IDAT files; 2 per sample) into a single GTC file per sample using the BPM file (defines strand, probe sequences and illumicode address) and the EGT file (defines the relationship between intensities and genotype calls). Files used for this data release are: GDA-8v1-0_A5.bpm, GDA-8v1-0_A1_ClusterFile.egt, gentrain v3, reference hg19 and gencall cutoff 0.15. The GDA array assays a total of 1,914,935 variant positions including 1,790,654 single-nucleotide variants, 44,172 indels, 9,935 intensity-only probes for CNV calling, and 70,174 duplicates (same position, different probes). Picard GtcToVcf is used to convert the GTC files to VCF format. Resulting VCF and IDAT files are submitted to the DRC for ingestion and further processing. The VCF file contains assay name, chromosome, position, genotype calls, quality score, raw and normalized intensities, B allele frequency and log *R* ratio values. Each genome centre is running the GDA array under Clinical Laboratory Improvement Amendments-compliant protocols. The GTC files are parsed and metrics are uploaded to in-house Laboratory Information Management System systems for QC review.

At batch level (each set of 96-well plates run together in the laboratory at one time), each genome centre includes positive control samples that are required to have >98% call rate and >99% concordance to existing data to approve release of the batch of data. At the sample level, the call rate and sex are the key QC determinants^41^. Contamination is also measured using BAFRegress^42^ and reported out as metadata. Any sample with a call rate below 98% is repeated one time in the laboratory. Genotyped sex is determined by plotting normalized *x* versus normalized *y* intensity values for a batch of samples. Any sample discordant with ‘sex at birth’ reported by the All of Us participant is flagged for further detailed review and repeated one time in the laboratory. If several sex-discordant samples are clustered on an array or on a 96-well plate, the entire array or plate will have data production repeated. Samples identified with sex chromosome aneuploidies are also reported back as metadata (XXX, XXY, XYY and so on). A final processing status of ‘pass’, ‘fail’ or ‘abandon’ is determined before release of data to the All of Us DRC. An array sample will pass if the call rate is >98% and the genotyped sex and sex at birth are concordant (or the sex at birth is not applicable). An array sample will fail if the genotyped sex and the sex at birth are discordant. An array sample will have the status of abandon if the call rate is <98% after at least two attempts at the genome centre.

Data from the arrays are used for participant return of genetic ancestry and non-health-related traits for those who consent, and they are also used to facilitate additional QC of the matched WGS data. Contamination is assessed in the array data to determine whether DNA re-extraction is required before WGS. Re-extraction is prompted by level of contamination combined with consent status for return of results. The arrays are also used to confirm sample identity between the WGS data and the matched array data by assessing concordance at 100 unique sites. To establish concordance, a fingerprint file of these 100 sites is provided to the Genome Centers to assess concordance with the same sites in the WGS data before CRAM submission.

### Genomic data curation

As seen in Extended Data Fig. 2, we generate a joint call set for all WGS samples and make these data available in their entirety and by sample subsets to researchers. A breakdown of the frequencies, stratified by computed ancestries for which we had more than 10,000 participants can be found in Extended Data Fig. 3. The joint call set process allows us to leverage information across samples to improve QC and increase accuracy.

#### Single-sample QC

If a sample fails single-sample QC, it is excluded from the release and is not reported in this document. These tests detect sample swaps, cross-individual contamination and sample preparation errors. In some cases, we carry out these tests twice (at both the Genome Center and the DRC), for two reasons: to confirm internal consistency between sites; and to mark samples as passing (or failing) QC on the basis of the research pipeline criteria. The single-sample QC process accepts a higher contamination rate than the clinical pipeline (0.03 for the research pipeline versus 0.01 for the clinical pipeline), but otherwise uses identical thresholds. The list of specific QC processes, passing criteria, error modes addressed and an overview of the results can be found in Supplementary Table 3.

#### Joint call set QC

During joint calling, we carry out additional QC steps using information that is available across samples including hard thresholds, population outliers, allele-specific filters, and sensitivity and precision evaluation. Supplementary Table 4 summarizes both the steps that we took and the results obtained for the WGS data. More detailed information about the methods and specific parameters can be found in the All of Us Genomic Research Data Quality Report^36^.

#### Batch effect analysis

We analysed cross-sequencing centre batch effects in the joint call set. To quantify the batch effect, we calculated Cohen’s *d* (ref. ^43^) for four metrics (insertion/deletion ratio, single-nucleotide polymorphism count, indel count and single-nucleotide polymorphism transition/transversion ratio) across the three genome sequencing centres (Baylor College of Medicine, Broad Institute and University of Washington), stratified by computed ancestry and seven regions of the genome (whole genome, high-confidence calling, repetitive, GC content of >0.85, GC content of <0.15, low mappability, the ACMG59 genes and regions of large duplications (>1 kb)). Using random batches as a control set, all comparisons had a Cohen’s *d* of <0.35. Here we report any Cohen’s *d* results >0.5, which we chose before this analysis and is conventionally the threshold of a medium effect size^44^.

We found that there was an effect size in indel counts (Cohen’s *d* of 0.53) in the entire genome, between Broad Institute and University of Washington, but this was being driven by repetitive and low-mappability regions. We found no batch effects with Cohen’s *d* of >0.5 in the ratio metrics or in any metrics in the high-confidence calling, low or high GC content, or ACMG59 regions. A complete list of the batch effects with Cohen’s *d* of >0.5 are found in Supplementary Table 8.

### Sensitivity and precision evaluation

To determine sensitivity and precision, we included four well-characterized control samples (four National Institute of Standards and Technology Genome in a Bottle samples (HG-001, HG-003, HG-004 and HG-005). The samples were sequenced with the same protocol as All of Us. Of note, these samples were not included in data released to researchers. We used the corresponding published set of variant calls for each sample as the ground truth in our sensitivity and precision calculations. We use the high-confidence calling region, defined by Genome in a Bottle v4.2.1, as the source of ground truth. To be called a true positive, a variant must match the chromosome, position, reference allele, alternate allele and zygosity. In cases of sites with multiple alternative alleles, each alternative allele is considered separately. Sensitivity and precision results are reported in Supplementary Table 5.

### Genetic ancestry inference

We computed categorical ancestry for all WGS samples in All of Us and made these available to researchers. These predictions are also the basis for population allele frequency calculations in the Genomic Variants section of the public Data Browser. We used the high-quality set of sites to determine an ancestry label for each sample. The ancestry categories are based on the same labels used in gnomAD^18^, the Human Genome Diversity Project (HGDP)^45^ and 1000 Genomes^1^: African (AFR); Latino/admixed American (AMR); East Asian (EAS); Middle Eastern (MID); European (EUR), composed of Finnish (FIN) and Non-Finnish European (NFE); Other (OTH), not belonging to one of the other ancestries or is an admixture; South Asian (SAS).

We trained a random forest classifier^46^ on a training set of the HGDP and 1000 Genomes samples variants on the autosome, obtained from gnomAD^11^. We generated the first 16 principal components (PCs) of the training sample genotypes (using the hwe_normalized_pca in Hail) at the high-quality variant sites for use as the feature vector for each training sample. We used the truth labels from the sample metadata, which can be found alongside the VCFs. Note that we do not train the classifier on the samples labelled as Other. We use the label probabilities (‘confidence’) of the classifier on the other ancestries to determine ancestry of Other.

To determine the ancestry of All of Us samples, we project the All of Us samples into the PCA space of the training data and apply the classifier. As a proxy for the accuracy of our All of Us predictions, we look at the concordance between the survey results and the predicted ancestry. The concordance between self-reported ethnicity and the ancestry predictions was 87.7%.

PC data from All of Us samples and the HGDP and 1000 Genomes samples were used to compute individual participant genetic ancestry fractions for All of Us samples using the Rye program. Rye uses PC data to carry out rapid and accurate genetic ancestry inference on biobank-scale datasets^47^. HGDP and 1000 Genomes reference samples were used to define a set of six distinct and coherent ancestry groups—African, East Asian, European, Middle Eastern, Latino/admixed American and South Asian—corresponding to participant self-identified race and ethnicity groups. Rye was run on the first 16 PCs, using the defined reference ancestry groups to assign ancestry group fractions to individual All of Us participant samples.

### Relatedness

We calculated the kinship score using the Hail pc_relate function and reported any pairs with a kinship score above 0.1. The kinship score is half of the fraction of the genetic material shared (ranges from 0.0 to 0.5). We determined the maximal independent set^41^ for related samples. We identified a maximally unrelated set of 231,442 samples (94%) for kinship scored greater than 0.1.

### LDL-C common variant GWAS

The phenotypic data were extracted from the Curated Data Repository (CDR, Control Tier Dataset v7) in the All of Us Researcher Workbench. The All of Us Cohort Builder and Dataset Builder were used to extract all LDL cholesterol measurements from the Lab and Measurements criteria in EHR data for all participants who have WGS data. The most recent measurements were selected as the phenotype and adjusted for statin use^19^, age and sex. A rank-based inverse normal transformation was applied for this continuous trait to increase power and deflate type I error. Analysis was carried out on the Hail MatrixTable representation of the All of Us WGS joint-called data including removing monomorphic variants, variants with a call rate of <95% and variants with extreme Hardy–Weinberg equilibrium values (*P* < 10^−15^). A linear regression was carried out with REGENIE^48^ on variants with a minor allele frequency >5%, further adjusting for relatedness to the first five ancestry PCs. The final analysis included 34,924 participants and 8,589,520 variants.

### Genotype-by-phenotype replication

We tested replication rates of known phenotype–genotype associations in three of the four largest populations: EUR, AFR and EAS. The AMR population was not included because they have no registered GWAS. This method is a conceptual extension of the original GWAS × phenome-wide association study, which replicated 66% of powered associations in a single EHR-linked biobank^49^. The PGRM is an expansion of this work by Bastarache et al., based on associations in the GWAS catalogue^50^ in June 2020 (ref. ^51^). After directly matching the Experimental Factor Ontology terms to phecodes, the authors identified 8,085 unique loci and 170 unique phecodes that compose the PGRM. They showed replication rates in several EHR-linked biobanks ranging from 76% to 85%. For this analysis, we used the EUR-, and AFR-based maps, considering only catalogue associations that were *P* < 5 × 10^−8^ significant.

The main tools used were the Python package Hail for data extraction, plink for genomic associations, and the R packages PheWAS and pgrm for further analysis and visualization. The phenotypes, participant-reported sex at birth, and year of birth were extracted from the All of Us CDR (Controlled Tier Dataset v7). These phenotypes were then loaded into a plink-compatible format using the PheWAS package, and related samples were removed by sub-setting to the maximally unrelated dataset (*n* = 231,442). Only samples with EHR data were kept, filtered by selected loci, annotated with demographic and phenotypic information extracted from the CDR and ancestry prediction information provided by All of Us, ultimately resulting in 181,345 participants for downstream analysis. The variants in the PGRM were filtered by a minimum population-specific allele frequency of >1% or population-specific allele count of >100, leaving 4,986 variants. Results for which there were at least 20 cases in the ancestry group were included. Then, a series of Firth logistic regression tests with phecodes as the outcome and variants as the predictor were carried out, adjusting for age, sex (for non-sex-specific phenotypes) and the first three genomic PC features as covariates. The PGRM was annotated with power calculations based on the case counts and reported allele frequencies. Power of 80% or greater was considered powered for this analysis.

### Reporting summary

Further information on research design is available in the Nature Portfolio Reporting Summary linked to this article.

## Online content

Any methods, additional references, Nature Portfolio reporting summaries, source data, extended data, supplementary information, acknowledgements, peer review information; details of author contributions and competing interests; and statements of data and code availability are available at 10.1038/s41586-023-06957-x.

### Supplementary information

Supplementary Information Supplementary Figs. 1–7, Tables 1–8 and Note.

Reporting Summary

Supplementary Dataset 1 Associations of ACKR1, HLA-DQB1 and TCF7L2 loci with all Phecodes stratified by genetic ancestry.

## Figures and Tables

**Extended Data Table 1:**
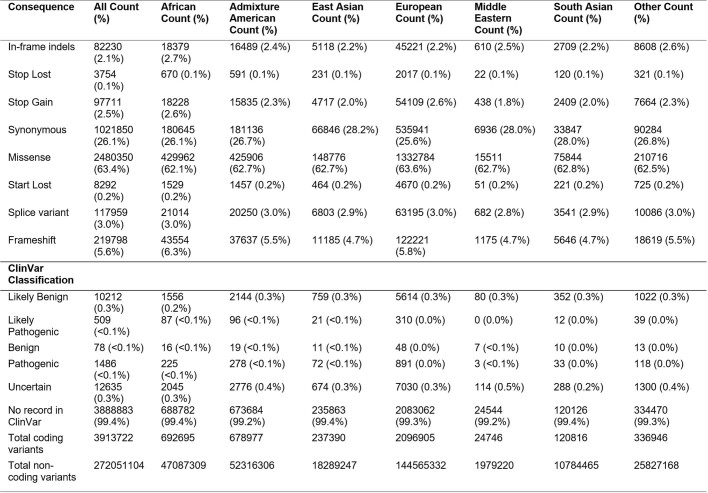
Coding consequence breakdown of All of Us Variants not previously described in dbSNP v153 in 245,388 short-read WGS samples

Percentages sum greater than zero, as variants can have multiple consequences depending on the transcript annotation.

**Extended Data Table 2:**
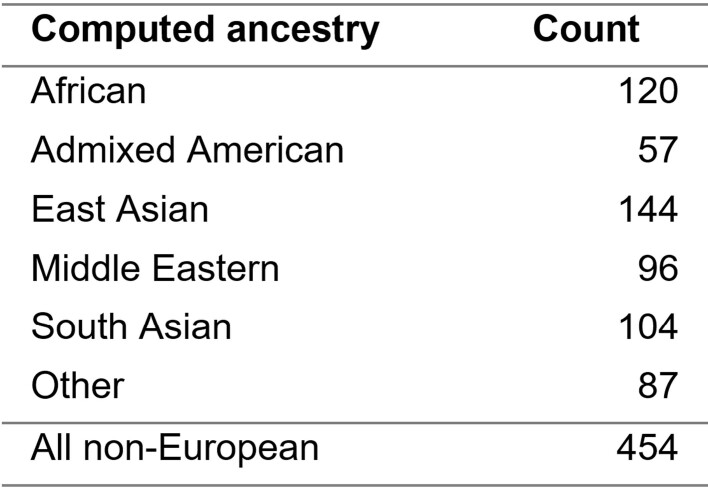
Number of coding variants common in non-EUR ancestry participants (minor allele frequency >1%) and not found in dbSNP v153 in 245,388 short-read WGS samples

**Extended Data Table 3:**
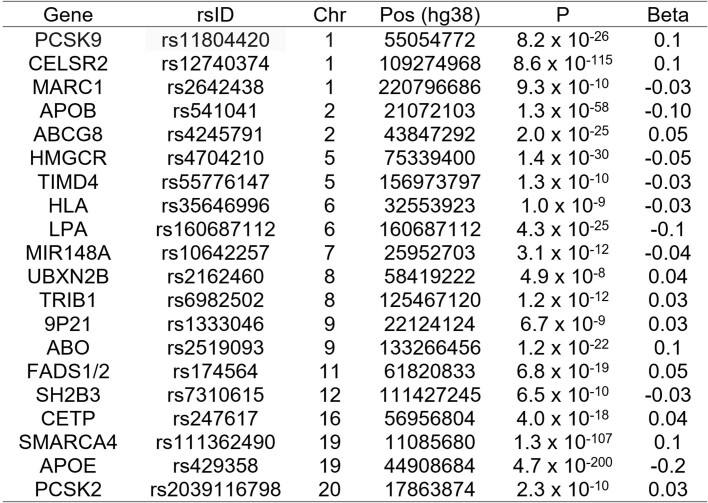
Genome-wide significant All of Us LDL-C GWAS loci in 91,749 All of Us individuals with one or more LDL-C measurements

**Extended Data Table 4:**
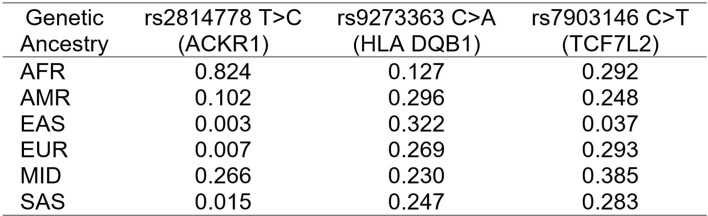
Allele Frequency by ancestral population of rs2814778, rs9273363 and rs7903146 in All of Us dataset

## Data Availability

The All of Us Research Hub has a tiered data access data passport model with three data access tiers. The Public Tier dataset contains only aggregate data with identifiers removed. These data are available to the public through Data Snapshots (https://www.researchallofus.org/data-tools/data-snapshots/) and the public Data Browser (https://databrowser.researchallofus.org/). The Registered Tier curated dataset contains individual-level data, available only to approved researchers on the Researcher Workbench. At present, the Registered Tier includes data from EHRs, wearables and surveys, as well as physical measurements taken at the time of participant enrolment. The Controlled Tier dataset contains all data in the Registered Tier and additionally genomic data in the form of WGS and genotyping arrays, previously suppressed demographic data fields from EHRs and surveys, and unshifted dates of events. At present, Registered Tier and Controlled Tier data are available to researchers at academic institutions, non-profit institutions, and both non-profit and for-profit health care institutions. Work is underway to begin extending access to additional audiences, including industry-affiliated researchers. Researchers have the option to register for Registered Tier and/or Controlled Tier access by completing the All of Us Researcher Workbench access process, which includes identity verification and All of Us-specific training in research involving human participants (https://www.researchallofus.org/register/). Researchers may create a new workspace at any time to conduct any research study, provided that they comply with all Data Use Policies and self-declare their research purpose. This information is made accessible publicly on the All of Us Research Projects Directory at https://allofus.nih.gov/protecting-data-and-privacy/research-projects-all-us-data.

## Code availability

The GVS code is available at https://github.com/broadinstitute/gatk/tree/ah_var_store/scripts/variantstore. The LDL GWAS pipeline is available as a demonstration project in the Featured Workspace Library on the Researcher Workbench (https://workbench.researchallofus.org/workspaces/aou-rw-5981f9dc/aouldlgwasregeniedsubctv6duplicate/notebooks).
