# Supplementary material for: Genomic data in the All of Us Research Program
Source: Nature. 2024 Feb 19;627(8003):340–6. doi: 10.1038/s41586-023-06957-x (PMC10937371; doi:10.1038/s41586-023-06957-x)
Supplement: Supplementary file 1 — Supplementary Figs. 1–7, Tables 1–8 and Note. [file 41586_2023_6957_MOESM1_ESM.docx]

# Supplementary Figures


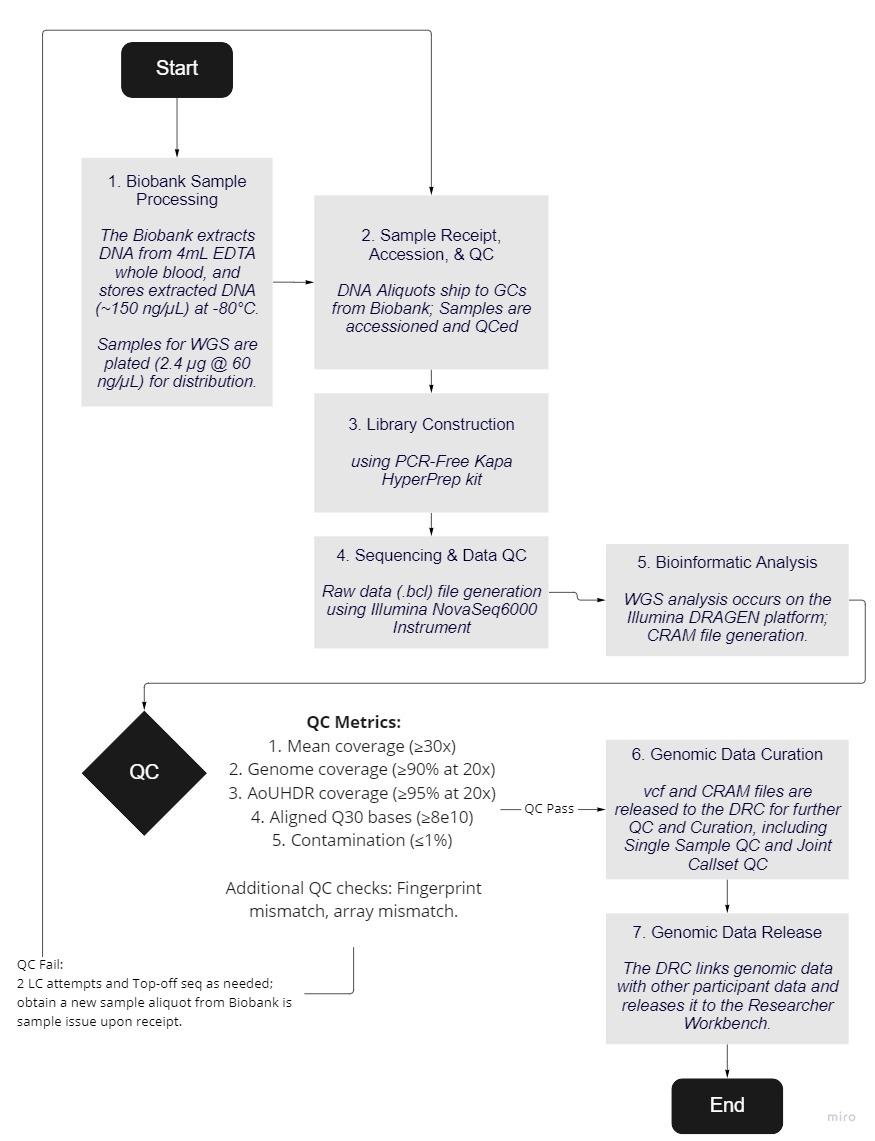


##

## Supplementary Fig 1. Overview of the All of Us Genome Sequencing workflow.

| a.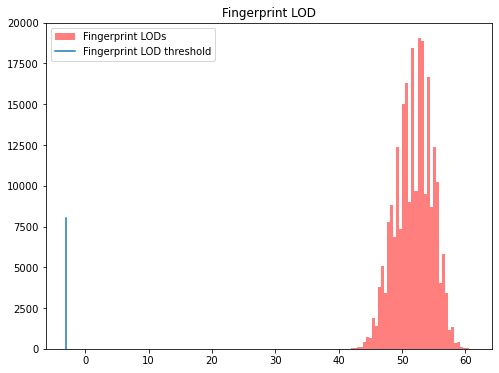 | b.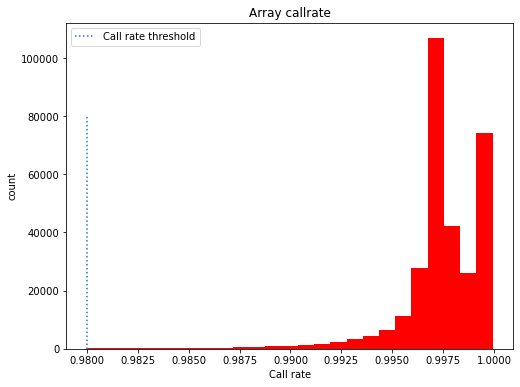 |
| --- | --- |
| c.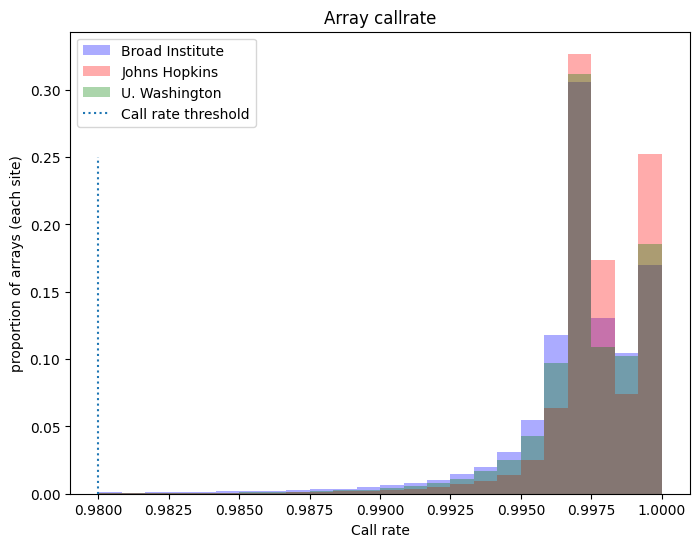 | d.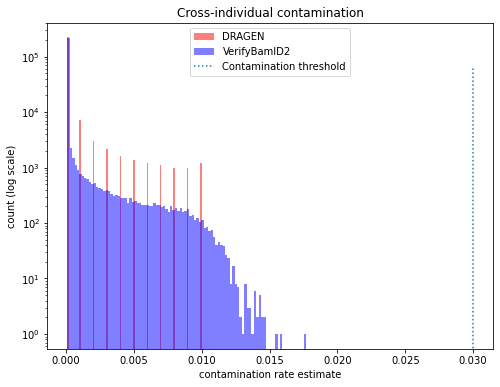 |

**Supplementary Fig 2.** **Single Sample quality control metrics.** **a**. Distribution of the Fingerprint LODs for 245,388 short-read WGS samples **b**. Histogram of the array call rate. Note that differences in call rates between males and females will cause a double peak in call rate frequencies, since sites on chrY will have a lower call rate for females **c.** Call rate across each Genome Center. The proportion (y-axis) is stratified by the genomic center. Median call rates for all centers were within 0.0005 for both males and females (determined by self-reported sex at birth). The 90th percentile call rates were within 0.0002 between all centers when stratified by self-reported sex at birth. **d.** WGS contamination estimates from both sources (DRAGEN and VerifyBamID2). DRAGEN rounds the contamination estimate to three decimal places. Note the log scale of the counts (y-axis). Over 89.0% and 91.4% of short-read WGS samples had contamination estimates lower than 1e-4 by VerifyBamID2 and DRAGEN, respectively. Any samples above the contamination threshold were not included in the dataset.


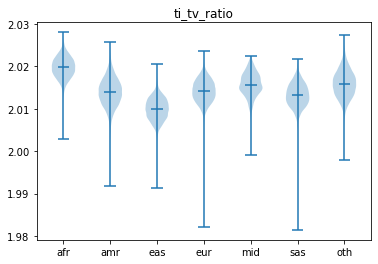


**Supplementary Fig 3.** **Metric distributions broken out by computed ancestry.** The metrics computed here are used to flag samples with outlying values. The list of flagged samples is available in the Researcher Workbench.


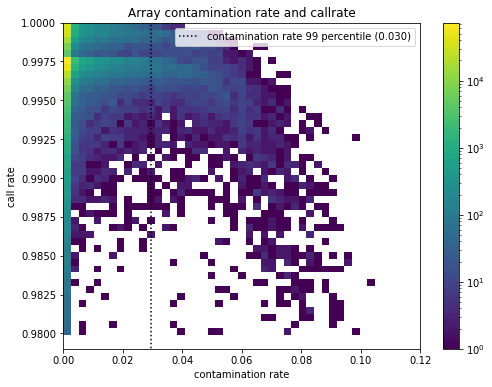


**Supplementary Fig 4. Histogram of the array contamination rate estimates vs call rate.** As the contamination rate increases, the call rate decreases.


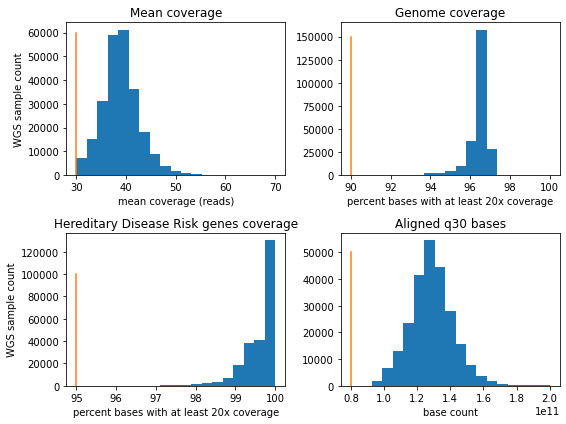


**Supplementary Fig 5. Coverage metrics for 245,394 WGS samples.** The orange line is the threshold for each metric. There are 281 samples (0.1%), with mean coverage greater than 70x, that are not included in the mean coverage (upper left) nor aligned q30 bases (lower right) plots. As expected, these samples were outliers in the number of aligned q30 bases (i.e., higher base count than samples with lower mean coverage).


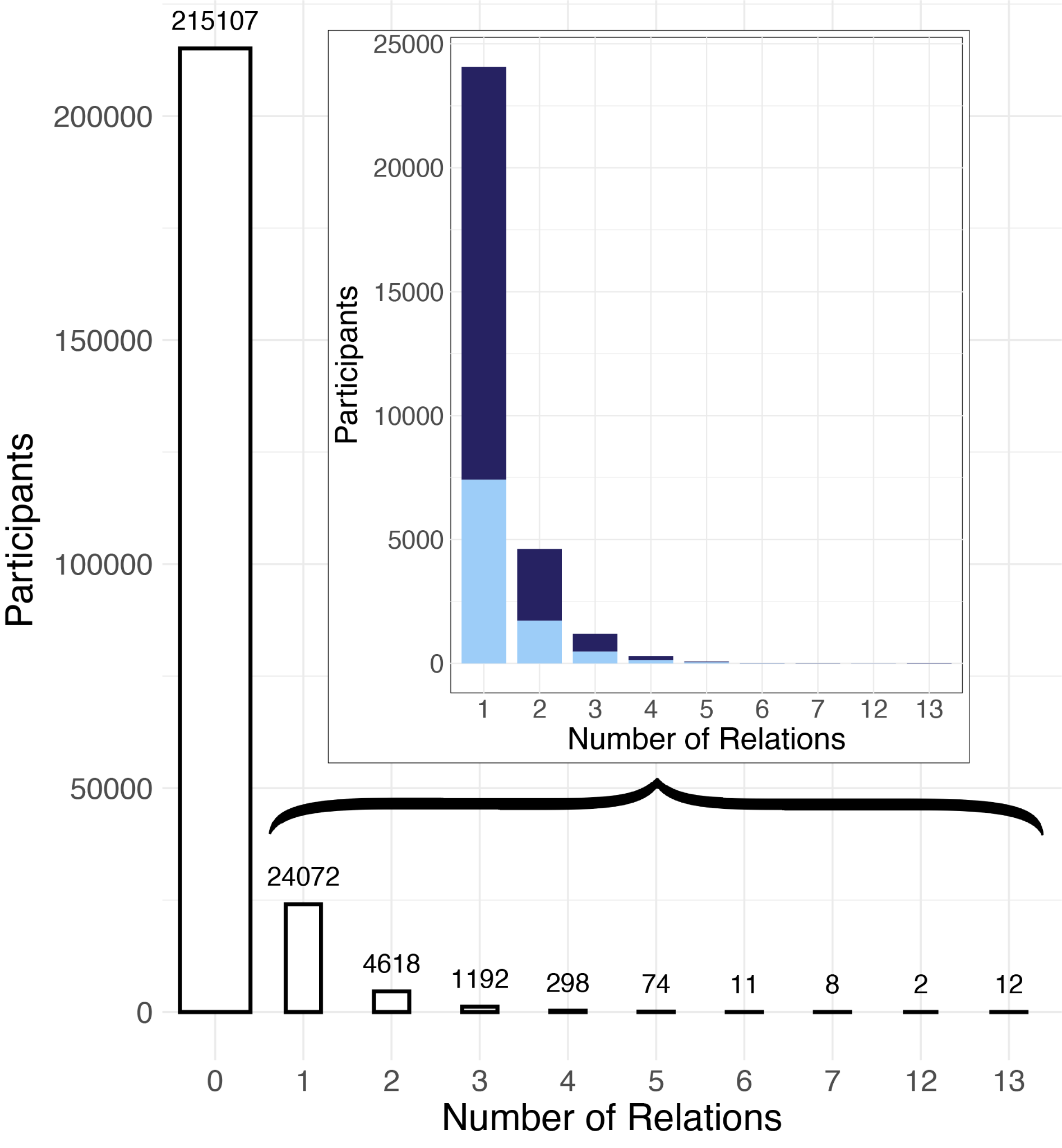


**Supplementary Fig 6. Relatedness in 245,388 short-read WGS samples.** Approximately ~14% of the *All of Us* cohort are (light blue) first- or (dark blue) second-degree relatives of one or more individuals in the cohort.


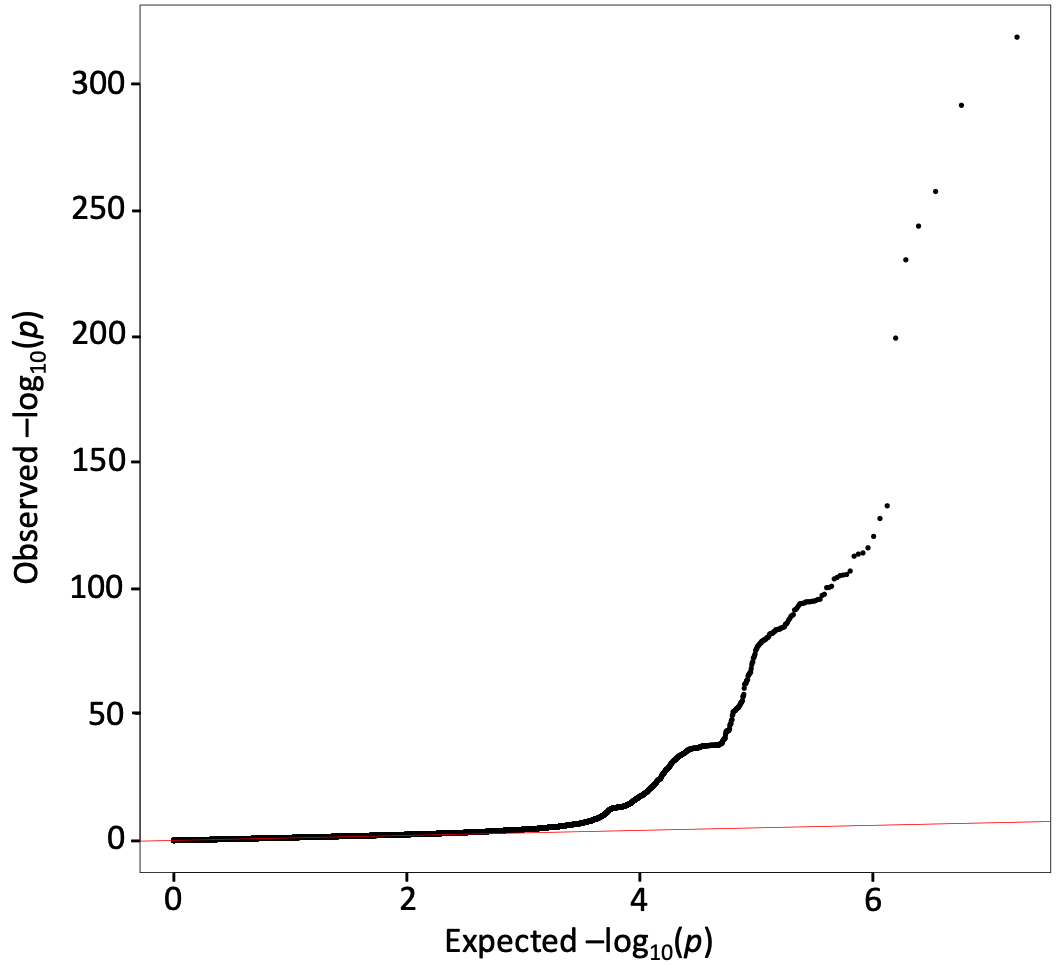


**Supplementary Fig. 7.** **Q-Q plot identifies minimal test-statistic inflation in LDL-C GWAS**, taken with a lambda value of 1.13, suggesting the phenotype and genotype data were appropriately quality controlled prior to running association tests.

**Supplementary Tables**

**Supplementary Table 1. *All of Us* participants stratified by Underrepresented in Biomedical Research (UBR) status in controlled tier**

| UBR Category | All Participants  (N, %) | | WGS  (N, %) | | Array  (N, %) | |
| --- | --- | --- | --- | --- | --- | --- |
| **Total Number** | 413,457 | -- | 245,388 | -- | 312,925 | -- |
| **At least one UBR** | 310,738 | 75.16% | 188,567 | 76.84% | 240,225 | 76.77% |
| **UBR Race/Ethnicity** | 179,022 | 43.30% | 112,673 | 45.92% | 142,099 | 45.41% |
| **UBR Sex Assigned at Birth** | 238 | 0.06% | 126 | 0.05% | 169 | 0.05% |
| **UBR Age** | 102,130 | 24.79% | 62,594 | 25.51% | 80,672 | 25.78% |
| **UBR Education** | 36,432 | 8.81% | 24,304 | 9.90% | 30,507 | 9.75% |
| **UBR Income** | 102,719 | 24.84% | 64,458 | 26.27% | 80,536 | 25.74% |
| **UBR Sexual and Gender Minorities** | 40,647 | 9.83% | 21,770 | 8.87% | 28,225 | 9.02% |

**Supplementary Table 2. Genome Center DRAGEN harmonized parameters for mapping and variant calling.**

| DRAGEN Parameter | Parameter Value | Description |
| --- | --- | --- |
| -f | n/a | Overwrite if output exists |
| -r <hg38-ref-dir> | <hg38-ref-dir> | The reference to use |
| --fastq-list | <path-to>/fastq_list.csv | A list of fastq files to use as input for this sample |
| --fastq-list-sample-id | <sampleID> | The sample ID to use for naming this sample |
| --output-directory | <output-dir> | The location of the final output files |
| --intermediate-results-dir | <int-results-dir> | The location to write intermediate outputs |
| --output-file-prefix | [CenterID]_[Biobankid_Sampleid]_[LocalID:optional]_[Rev#] | Standardized naming prefix for each output file |
| --enable-variant-caller | true | Turn on variant call outputs |
| --enable-duplicate-marking | true | Mark duplicate reads during alignment |
| --enable-map-align | true | Produce an alignment from unaligned read input |
| --enable-map-align-output | true | Store the output of the alignment |
| --output-format | CRAM | Store the alignment as a CRAM file |
| --vc-hard-filter | DRAGENHardQUAL:all:QUAL<5.0;LowDepth:all:DP<=1' | This parameter setting changes the threshold on the quality to 5. |
| --vc-frd-max-effective-depth | 40 | Setting this parameter puts a limit on the penalty value that is applied for variant calls that deviate from the expected 50% allele fraction for heterozygous variants. |
| --qc-cross-cont-vcf | <path-to/SNP_NCBI_GRCh38.vcf> | Marker sites to use for contamination estimation |
| --qc-coverage-region-1 | <path-to/wgs_coverage_regions.bed> | Regions to use for coverage analysis (whole genome) |
| --qc-coverage-reports-1 | cov_report | The type of reports requested for qc- coverage-region-1 |
| --qc-coverage-region-2 | <path-to/HDRR_regions.bed> | Regions to use for coverage analysis (HDRR reportable regions) |
| --qc-coverage-reports-2 | cov_report | The type of reports requested for qc- coverage-region-2 |
| --qc-coverage-region-3 | <path-to/PGx_regions.bed> | Regions to use for coverage analysis (PGx reportable regions) |
| --qc-coverage-reports-3 | cov_report | The type of reports requested for qc- coverage-region-3 |

**Supplementary Table 3. Data and Research Center Single Sample QC summary**

| QC process | Data types | Passing criteria | Error modes addressed | Result |
| --- | --- | --- | --- | --- |
| **Fingerprint concordance** | WGS (uses Arrays) | log-likelihood ratio > -3 | -Sample swaps  -Large amount of sample contamination | All array and WGS sample pairs are concordant. |
| **Sex concordance** | WGS and Arrays | Sex call is concordant with self-reported sex at birth. OR  Self-reported sex at birth reported as “Other” or was not reported | -Sample swaps | All array and WGS samples are concordant. |
| **Call rate** | Arrays | > 0.98 (> 98%) | -Sample contamination  -Sample preparation error | All array samples meet the threshold. |
| **Cross-individual contamination rate** | WGS and Arrays | WGS: < 0.03 (< 3%)  Arrays: None (Reported only) | Sample contamination from another individual | All WGS samples meet the threshold.  For arrays, we only report the contamination rate, but do not filter array samples, since the call rate is a proxy for high levels of contamination.  WGS samples with corresponding arrays that have a contamination rate above 10% were not released. |
| **Coverage** | WGS | ≥ 30x mean coverage  ≥ 90% of bases at 20x coverage  ≥8e10 aligned Q30 Bases  ≥ 95% at 20x in regions of the 59 *All of Us* Hereditary Disease Risk genes | -Sample preparation error  -Poor sensitivity and precision of variant calling | All WGS samples meet the thresholds. |

**Supplementary Table 4. Joint call set QC summary**

| QC process | Variant/sample | Error modes addressed | Result |
| --- | --- | --- | --- |
| Hard Thresholds | sample | Extremely noisy samples | No samples flagged. |
| Population Outlier | sample | Noisy samples | 551 samples flagged (0.2%).  Based on regressing out the PCAs from callset metrics, such as snp_count. |
| Hard Threshold Filters | variant | Artifacts that cannot be detected in a single sample | This has a simple implementation with high precision, which saves compute for downstream variant filtering.  59,496,403 were filtered  972,115,272 were not filtered |
| Allele-Specific VariantQualityScoreRecalibration (AS-VQSR) | variant | Artifacts that cannot be detected in a single sample |  |
| Sensitivity and Precision Evaluation | both | Poor variant detection | We use the high-confidence region calls from Genomes in a Bottle as a ground truth for calls for evaluation. |

**Supplementary Table 5. Sensitivity and precision measurements for control samples using the *All of Us* sequencing protocol**

| Variant type | NIST ID | 1000 Genomes ID | Sensitivity | Precision |
| --- | --- | --- | --- | --- |
| SNV | HG-001 | NA12878 | 0.995 | >0.999 |
|  | HG-003 | NA24149 | 0.988 | >0.999 |
|  | HG-004 | NA24143 | 0.988 | >0.999 |
|  | HG-005 | NA24631 | 0.989 | >0.999 |
| Indel | HG-001 | NA12878 | 0.987 | 0.996 |
|  | HG-003 | NA24149 | 0.985 | 0.997 |
|  | HG-004 | NA24143 | 0.986 | 0.998 |
|  | HG-005 | NA24631 | 0.994 | 0.999 |

**Supplementary Table 6.** **OMOP concepts collected from recruitment sites in the *All of Us Research Program*.**

| Standardized clinical data tables | Standardized vocabularies |
| --- | --- |
| [PERSON](https://github.com/OHDSI/CommonDataModel/wiki/PERSON)  [OBSERVATION_PERIOD](https://github.com/OHDSI/CommonDataModel/wiki/OBSERVATION_PERIOD)  [VISIT_OCCURRENCE](https://github.com/OHDSI/CommonDataModel/wiki/VISIT_OCCURRENCE)  [VISIT_DETAIL](https://github.com/OHDSI/CommonDataModel/wiki/VISIT_DETAIL)  [CONDITION_OCCURRENCE](https://github.com/OHDSI/CommonDataModel/wiki/CONDITION_OCCURRENCE)  [DEATH](https://github.com/OHDSI/CommonDataModel/wiki/DEATH)  [DRUG_EXPOSURE](https://github.com/OHDSI/CommonDataModel/wiki/DRUG_EXPOSURE)  [PROCEDURE_OCCURRENCE](https://github.com/OHDSI/CommonDataModel/wiki/PROCEDURE_OCCURRENCE)  [DEVICE_EXPOSURE](https://github.com/OHDSI/CommonDataModel/wiki/DEVICE_EXPOSURE)  [MEASUREMENT](https://github.com/OHDSI/CommonDataModel/wiki/MEASUREMENT)  [SURVEY_CONDUCT](https://github.com/OHDSI/CommonDataModel/wiki/SURVEY_CONDUCT)  [OBSERVATION](https://github.com/OHDSI/CommonDataModel/wiki/OBSERVATION)  [SPECIMEN](https://github.com/OHDSI/CommonDataModel/wiki/SPECIMEN)  [FACT_RELATIONSHIP](https://github.com/OHDSI/CommonDataModel/wiki/FACT_RELATIONSHIP) | [CONCEPT](https://github.com/OHDSI/CommonDataModel/wiki/CONCEPT)  [VOCABULARY](https://github.com/OHDSI/CommonDataModel/wiki/VOCABULARY)  [DOMAIN](https://github.com/OHDSI/CommonDataModel/wiki/DOMAIN)  [CONCEPT_CLASS](https://github.com/OHDSI/CommonDataModel/wiki/CONCEPT_CLASS)  [CONCEPT_RELATIONSHIP](https://github.com/OHDSI/CommonDataModel/wiki/CONCEPT_RELATIONSHIP)  [RELATIONSHIP](https://github.com/OHDSI/CommonDataModel/wiki/RELATIONSHIP)  [CONCEPT_SYNONYM](https://github.com/OHDSI/CommonDataModel/wiki/CONCEPT_SYNONYM)  [CONCEPT_ANCESTOR](https://github.com/OHDSI/CommonDataModel/wiki/CONCEPT_ANCESTOR)  [DRUG_STRENGTH](https://github.com/OHDSI/CommonDataModel/wiki/DRUG_STRENGTH) |

**Supplementary Table 7.** **Genome Center specific components of WGS production pipeline.**

|  | **Genome Centers** | | |
| --- | --- | --- | --- |
|  | **Baylor** | **Broad** | **University of Washington** |
| **Sample Accessioning & QC** | | | |
| **Quantitation** | Picogreen (Synergy) or DropQuant | Picogreen | Invitrogen Quant-it |
| **Automation / Liquid Handler** | Biomek FXp | Dynamic Devices Lynx | SPT LabTech Mosquito and Perkin Elmer Janus |
| **Library Construction** | | | |
| **Library prep** | PCR Free Kapa HyperPrep | PCR Free Kapa HyperPrep | PCR Free Kapa HyperPrep |
| **Barcodes** | 96  8-bp Illumina TruSeq DNA UD Indices | 8-bp unique dual indices (Roche) | 576  10-bp unique dual indices |
| **Automation / Liquid Handler** | Biomek FXp | Agilent Bravos | Perkin Elmer Janus |
| **Sonicator** | Covaris E220 | Covaris LE220-Plus | Covaris LE220 |
| **Library QC - Quantitation** | AB QuantStudio 6 Flex | Viia7 qPCR machine | Biorad CFX384 |
| **Library QC - Size estimation** | Agilent Bioanalyzer 2100 or Agilent Fragment Analyzer | Agilent Bioanalyzer 2100 or Agilent Fragment Analyzer | Agilent Fragment Analyzer |
| **Whole Genome Sequencing (WGS)** | | | |
| **Sequencer** | NovaSeq 6000 | NovaSeq 6000 | NovaSeq 6000 |
| **Multiplexing & Sequencing Strategy** | 2 pooling methods:  - 24-plex on S4 flowcell  - calibration pool with re-pool: 75-plex on 12 lanes of S4 flowcell | - 24-plex pool on S4 flowcell  - adaptive pooling | - 192-plex pool for NovaSeq XP QC run  - 26-plex pool on S4 flowcell |

**Supplementary Table 8.** **Batch effects across sequencing centers.**

| **Genome Center 1** | **Genome Center 2** | **Metric** | **% Difference** | **Cohen’s d (Effect Size)** | | **Computed Ancestry** |
| --- | --- | --- | --- | --- | --- | --- |
| Whole genome metrics with effect size greater than 0.5 | | | | | | |
| Broad | UW | Indel Count | 0.45 | 0.53 | | EAS |
| Broad | UW | Indel Count | 0.49 | 0.51 | | EUR |
| Low mappability metrics with effect size greater than 0.5 | | | | | | |
| Baylor | Broad | Indel Count | -2.49 | 0.87 | | AFR |
| Broad | UW | Indel Count | 2.91 | 1.05 | | AFR |
| Baylor | UW | SNP Count | 1.69 | 0.52 | | AMR |
| Broad | UW | SNP Count | 2.14 | 0.64 | | AMR |
| Baylor | Broad | Indel Count | -2.49 | 0.65 | | AMR |
| Baylor | UW | Indel Count | 2.22 | 0.65 | | AMR |
| Broad | UW | Indel Count | 4.71 | 1.36 | | AMR |
| Broad | UW | SNP Count | 1.12 | 0.74 | | EAS |
| Baylor | Broad | Indel Count | -2.44 | 1.11 | | EAS |
| Baylor | UW | Indel Count | 1.22 | 0.57 | | EAS |
| Broad | UW | Indel Count | 3.65 | 1.66 | | EAS |
| Broad | UW | SNP Count | 0.97 | 0.56 | | EUR |
| Baylor | Broad | Indel Count | -2.5 | 1.04 | | EUR |
| Baylor | UW | Indel Count | 1.21 | 0.52 | | EUR |
| Broad | UW | Indel Count | 3.71 | 1.51 | | EUR |
| Broad | UW | SNP Count | 1.07 | 0.59 | | SAS |
| Baylor | Broad | Indel Count | -2.4 | 0.96 | | SAS |
| Broad | UW | Indel Count | 3.36 | 1.34 | | SAS |
| Identified batch effects for the tandem repeat regions of the genome | | | | | | |
| Broad | UW | SNP Count | 0.53 | 0.57 | | EAS |
| Broad | UW | SNP Count | 0.61 | 0.58 | | EUR |
| Broad | UW | SNP Count | 0.66 | 0.52 | | SAS |
| Broad | UW | Indel Count | 1.7 | 0.52 | | AMR |
| Broad | UW | Indel Count | 0.51 | 0.57 | | EAS |
| Broad | UW | Indel Count | 0.57 | 0.56 | | EUR |
| Broad | UW | Indel Count | 0.72 | 0.51 | | SAS |
| Batch effects seen in segmental duplication regions | | | | | | |
| Broad | UW | Indel Count | 3.06 | | 0.72 | AMR |
| Baylor | Broad | Indel Count | -1.66 | | 0.55 | EAS |
| Broad | UW | Indel Count | 1.71 | | 0.59 | EAS |
| Broad | UW | Indel Count | 1.72 | | 0.53 | SAS |

### **Supplementary Note**

### **Representation of Groups Historically Underrepresented in Biomedical Research (UBR)**

Program definitions of several UBR categories of focus are listed below.^49^

1. Race & Ethnicity: Individuals who identify as other than White and non-Hispanic (i.e., Asian; Black, African American, or African; Hispanic, Latino, or Spanish; Middle Eastern or North African; or Native Hawaiian or other Pacific Islander) Information from participants who self-identify as American Indian, or Alaska Native will be available in a future data release.
2. Sex Assigned at Birth, Sexual Orientation, Gender Identity (SOGI): Individuals who identify as neither male or female; individuals who identify as gender variant, non-binary, transgender, or something else other than man or woman; individuals who identify as asexual, bisexual, gay or lesbian, or something else other than straight.
3. Age: Children 17 or younger and adults 65 or older. (Note: *All of Us* is not currently enrolling minors, though this is a future goal.)
4. Educational Attainment: Individuals with less than a high school degree or equivalent.
5. Annual Household Income: Individuals with household incomes equal to or below 200% of the Federal Poverty Level.
6. Geography: Individuals who reside in rural and non-metropolitan areas.
7. *Access to care: Individuals who have not had a needed medical visit in the past 12 months or cannot easily obtain or pay for medical care as needed. This category will be available in a future data release.
8. *Disability: Individuals with either a physical or cognitive disability. This category will be available in a future data release.
