# Supplementary material for: Genomic data in the All of Us Research Program
Source: Nature. 2024 Feb 19;627(8003):340–6. doi: 10.1038/s41586-023-06957-x (PMC10937371; doi:10.1038/s41586-023-06957-x)
Supplement: Supplementary file 2 — Reporting Summary [file 41586_2023_6957_MOESM2_ESM.pdf]

nature portfolio

Corresponding author(s): Bick
Last updated by author(s): Nov 22, 2023

Reporting Summary

Nature Portfolio wishes to improve the reproducibility of the work that we publish. This form provides structure for consistency and transparency in reporting. For further information on Nature Portfolio policies, see our Editorial Policies and the Editorial Policy Checklist.

Statistics

For all statistical analyses, confirm that the following items are present in the figure legend, table legend, main text, or Methods section.

n/a	Confirmed	
☐	☒	The exact sample size (*n*) for each experimental group/condition, given as a discrete number and unit of measurement
☒	☐	A statement on whether measurements were taken from distinct samples or whether the same sample was measured repeatedly
☐	☒	The statistical test(s) used AND whether they are one- or two-sided<br>*Only common tests should be described solely by name; describe more complex techniques in the Methods section.*
☐	☒	A description of all covariates tested
☐	☒	A description of any assumptions or corrections, such as tests of normality and adjustment for multiple comparisons
☐	☒	A full description of the statistical parameters including central tendency (e.g. means) or other basic estimates (e.g. regression coefficient) AND variation (e.g. standard deviation) or associated estimates of uncertainty (e.g. confidence intervals)
☐	☒	For null hypothesis testing, the test statistic (e.g. *F*, *t*, *r*) with confidence intervals, effect sizes, degrees of freedom and *P* value noted<br>*Give P values as exact values whenever suitable.*
☒	☐	For Bayesian analysis, information on the choice of priors and Markov chain Monte Carlo settings
☒	☐	For hierarchical and complex designs, identification of the appropriate level for tests and full reporting of outcomes
☒	☐	Estimates of effect sizes (e.g. Cohen's *d*, Pearson's *r*), indicating how they were calculated

Our web collection on statistics for biologists contains articles on many of the points above.

Software and code

Policy information about availability of computer code

Data collection	N/A
Data analysis	The genomic variant store code is available at:<br>https://github.com/broadinstitute/gatk/tree/ah_var_store/scripts/variantstore<br>The LDL GWAS pipeline is available as a demonstration project in the Featured Workspace Library on the Researcher Workbench:<br>https://workbench.researchallofus.org/workspaces/aou-rw-5981f9dc/aouldlgwasregeniedsubctv6duplicate/notebooks

For manuscripts utilizing custom algorithms or software that are central to the research but not yet described in published literature, software must be made available to editors and reviewers. We strongly encourage code deposition in a community repository (e.g. GitHub). See the Nature Portfolio guidelines for submitting code & software for further information.

Data

Policy information about availability of data

All manuscripts must include a data availability statement. This statement should provide the following information, where applicable:
- Accession codes, unique identifiers, or web links for publicly available datasets
- A description of any restrictions on data availability
- For clinical datasets or third party data, please ensure that the statement adheres to our policy

The All of Us Research Hub has a tiered data access data passport model with three data access tiers. The Public Tier dataset contains only aggregate data with

identifiers removed. These data are available to the public through Data Snapshots (https://www.researchallofus.org/data-tools/data-snapshots/) and the public Data Browser (https://databrowser.researchallofus.org/). The Registered Tier curated dataset contains individual-level data, available only to approved researchers on the Researcher Workbench. The Registered Tier currently includes data from electronic health records (EHRs), wearables, and surveys, as well as physical measurements taken at the time of participant enrollment. The Controlled Tier dataset contains all data in the Registered Tier and additionally genomic data in the form of whole genome sequencing (WGS) and genotyping arrays, previously suppressed demographic data fields from EHRs and surveys, and unshifted dates of events. Registered Tier and Controlled Tier data are currently available to researchers at academic institutions, non-profit institutions, and both non-profit and for-profit healthcare institutions. Work is underway to begin extending access to additional industry affiliated researchers. Researchers have the option to register for Registered Tier and/or Controlled Tier access by completing the All of Us Researcher Workbench access process which includes identity verification and All of Us-specific human subjects training (https://www.researchallofus.org/register/). Researchers may create a new workspace at any time to conduct any research study, provided that they comply with all Data Use Policies and self-declare their research purpose. This information is made accessible publicly on the All of Us Research Projects Directory at https://allofus.nih.gov/protecting-data-and-privacy/research-projects-all-us-data

Human research participants

Policy information about studies involving human research participants and Sex and Gender in Research.

Reporting on sex and gender	Both sex assigned at birth and self reported gender of individuals was collected. Sex assigned at birth was used for all relevant analyses, including only individuals where the genetically inferred sex matched sex assigned at birth.
Population characteristics	Adults 18 years and older who have the capacity to consent and currently reside in the U.S. or a U.S. territory were eligible.
Recruitment	Recruitment of the All of Us Research Program was described in detail in "The “All of Us” Research Program", NEJM 2019; briefly individuals were recruited through direct participant enrollment or recruitment at one of >340 locations at US healthcare provider organizations or federally qualified community health centers.
Ethics oversight	Informed consent for all participants is conducted in person or through an eConsent platform that includes primary consent, HIPAA Authorization for Research EHRs, and Consent for Return of Genomic Results. The protocol was reviewed by the Institutional Review Board (IRB) of the All of Us Research Program. The All of Us IRB follows the regulations and guidance of the NIH Office for Human Research Protections for all studies, ensuring that the rights and welfare of research participants are overseen and protected uniformly.

Note that full information on the approval of the study protocol must also be provided in the manuscript.

Field-specific reporting

Please select the one below that is the best fit for your research. If you are not sure, read the appropriate sections before making your selection.

☒ Life sciences ☐ Behavioural & social sciences ☐ Ecological, evolutionary & environmental sciences

For a reference copy of the document with all sections, see nature.com/documents/nr-reporting-summary-flat.pdf

Life sciences study design

All studies must disclose on these points even when the disclosure is negative.

Sample size	No pre-determined sample size was calculated for these analyses.
Data exclusions	No data or individuals with successful generation of genome sequencing data were excluded from these analyses.
Replication	Replication of the LDL cholesterol GWAS study was performed with the NHLBI TOPMed study
Randomization	There was no randomization.
Blinding	There was no blinding.

Reporting for specific materials, systems and methods

We require information from authors about some types of materials, experimental systems and methods used in many studies. Here, indicate whether each material, system or method listed is relevant to your study. If you are not sure if a list item applies to your research, read the appropriate section before selecting a response.

Materials & experimental systems

n/a	Involved in the study
☒	☐ Antibodies
☒	☐ Eukaryotic cell lines
☒	☐ Palaeontology and archaeology
☒	☐ Animals and other organisms
☒	☐ Clinical data
☒	☐ Dual use research of concern

Methods

n/a	Involved in the study
☒	☐ ChIP-seq
☒	☐ Flow cytometry
☒	☐ MRI-based neuroimaging
